# Temporal trends and spatial differences of trace elements in osprey eggs in Finland

**DOI:** 10.1007/s10661-026-15460-5

**Published:** 2026-05-23

**Authors:** Matti Viluksela, Pertti Saurola, Juhani Koivusaari, Matts Finnlund, Timo Sara-Aho, Anders Bignert, Jouni T. Tuomisto, Hannu Kiviranta, Matti Verta

**Affiliations:** 1 School of Pharmacy (Toxicology) and Department of Environmental and Biological Sciences, University of Eastern Finland, Kuopio, Finland; 2 Department of Public Health, Finnish Institute for Health and Welfare (THL), P.O. Box 95, 70701 Kuopio, Finland; 3 Ringing Centre, Finnish Museum of Natural History, University of Helsinki, P.O. Box 17, 00014 Helsinki, Finland; 4 Finnish Environment Institute, Helsinki, Finland; 5 Yibin Research Base of the Key Laboratory of Yangtze River Water Environment of the Ministry of Education, Yibin University, Yibin, 644000 Sichuan Province China; 6 Current address: Kausal, Kuopio, Finland

**Keywords:** Mercury, Cadmium, Lead, Metals, Raptor

## Abstract

Time trends and regional differences of total mercury (THg), methylmercury (MeHg), cadmium (Cd), chromium (Cr), copper (Cu), lead (Pb), selenium (Se), zinc (Zn), palladium (Pd), platinum (Pt), and rhodium (Rh) concentrations were studied and toxicological significance of the observed levels was assessed in unhatched osprey (*Pandion haliaetus*) eggs collected by bird ringers in 1972–2005 from 2 to 3 areas in Finland. The study areas were Northern Quark in the Gulf of Bothnia, Baltic Sea, and eutrophic-dystrophic Lake Vanajanselkä, which is affected by industrial emissions. Significant declining trends were found for Pb (average annual decrease 6.9%), Cr (2.8%), MeHg (1.9%), and THg (1.3%), with slightly faster decrease in Northern Quark (7.5%, 3.4%, 3.1%, and 1.8%, respectively) compared to Lake Vanajanselkä (6.9%, 2.6%, 1.2%, and 0.8%, respectively). Metal concentrations in eggs did not exhibit significant regional differences. This pattern aligns with metal concentrations in prey fish from the same areas underscoring the role of long-range atmospheric deposition as the source of exposure. Among the studied metals, only a few eggs with highest THg and Pb concentrations exceeded toxicological thresholds: THg threshold for moderate injury (0.3 µg/g ww) 6.1%, high injury (0.7 µg/g ww) 0.88%, Pb tentative threshold (1.2 µg/g ww) 2.6%. These levels could have contributed to decreased reproductive success. The gradual recovery of the Finnish osprey population during the study period paralleled with declining levels of metals and persistent organic pollutants. This study highlights the value of environmental specimen collections and long-term time series in an apex predator for monitoring aquatic environments.

Clinical trial number: not applicable.

**Supplementary Information:**

The online version contains supplementary material available at 10.1007/s10661-026-15460-5.

## Introduction

Metals are naturally occurring elements found throughout the Earth’s crust; many are essential for life, but anthropogenic activities can increase their release, accumulation, and biomagnification, leading to harmful effects on the environment, wildlife, and humans. Although international treaties such as the Basel Convention (1989) and the Rotterdam Convention (1998) have decreased the release of hazardous metals to the environment and reduced their risks, continued surveillance is required to monitor their distribution, levels and trends. The value of more efficient use of existing environmental specimen collections from apex predators over temporal and spatial scales has been recently highlighted in advancing prioritization and risk assessment of chemicals (Movalli et al., 2022; Treu et al., 2022).

Atmospheric emissions from energy production, municipal waste incineration, coal combustion, and chloralkali industries for production of chlorine have historically been the dominant contributors to Hg inputs in Nordic ecosystems (HELCOM, 2018; Jaakkonen, 2011). Elemental chlorine was used in Finland for bleaching of pulp until 1992, and Hg was used as a slimicide in the paper and pulp industry until 1968. Hg and its alkyl derivatives have also been used as an agricultural seed dressing in the past. Hg evaporates easily and the chemically stable vapor is globally distributed. Hg is methylated by bacteria to MeHg, which is lipophilic, more readily biomagnified, and much more toxic than inorganic Hg (Ufelle & Barchowsky, 2019). The proportion of MeHg of total Hg (THg) increases with the trophic level in the food web from about 15% in phytoplankton to > 90% in fish-eating birds (Ackerman et al., 2013; Watras & Bloom, 1992). MeHg is a potent neurotoxicant causing e.g. paresthesia, ataxia, vision and hearing loss, impaired brain development, and behavioral alterations. In birds, reproduction is among the most sensitive endpoints of MeHg toxicity (Ackerman et al., 2024; Fuchsman et al., 2016), and egg THg concentrations > 1 µg/g wet weight (ww) are generally associated with impaired hatchability and embryonic mortality (Scheuhammer et al., 2007).

In addition to long-range transport, local industrial point sources contribute to deposition of Cd and Pb (Skjelkvåle et al., 2001; Verta et al., 2010). The main source of Cd has been coal burning and that of Pb the use of leaded gasoline. Both Cd and Pb can bioaccumulate and be transferred in the food web. Toxic effects of Cd include kidney and bone toxicity and those of Pb neurotoxicity, especially developmental neurotoxicity (Ufelle & Barchowsky, 2019). In birds, exposure to high Cd concentrations was linked to renal tubular damage, skeletal toxicity (Larison et al., 2000), decreased egg production, testicular damage, and altered behavioral responses (Wayland & Scheuhammer, 2011). Exposure to Pb results in decreased clutch and egg size, depressed growth, mortality of embryos and nestlings, and deficits in behavior affecting survival (Burger, 1995).

Of the essential metals with toxic potential Cr, Cu, and Zn have locally important industrial and other anthropogenic sources of emission while the long-range transport is less significant in the Nordic countries (Poikolainen et al., 2004; Steinnes et al., 2011). Concentrations of Se, Cu, and Zn in the body are physiologically regulated, but their levels in tissues still reflect ambient environmental concentrations (Ufelle & Barchowsky, 2019). Se-dependent enzymes protect cells against oxidative damage, and Se has been shown to reduce toxicity of Hg (Adams & Duguay, 2025). Natural Se concentrations are generally higher in seawater than in freshwater (Amouroux et al., 2001). The main anthropogenic Se emissions in Finland are nonferrous metal production and combustion of fossil fuels (Wang et al., 1993). Regardless of emissions and atmospheric deposition, surface waters, rocks, and soils in Finland, Sweden, and Norway have naturally low Se concentrations compared to other European regions. Therefore, fertilizers have been fortified in Finland with sodium selenate since 1985, but no obvious effects have been found on aquatic ecosystems (Alfthan et al., 2015; Varo et al., 1994).

The osprey (*Pandion haliaetus*) is a useful bioindicator of aquatic contamination due to its strict piscivory and high trophic position (Grove et al., 2009). In Finland, a long-term monitoring program initiated in the early 1970 s provides access to archived unhatched eggs suitable for retrospective contaminant analyses (Saurola, 1980, 2025). Although ospreys nesting in Finland overwinter in West Africa, earlier work has shown that contaminant burdens in eggs and feathers primarily reflect exposures near the breeding sites (Elliott et al., 1998, 2007; Odsjö et al., 2004; Viluksela et al., 2024). Eggs are an ideal low-invasive tissue for monitoring exposure to toxicologically relevant metals in birds, because reproductive and developmental effects are among the most sensitive endpoints of toxicity (Scheuhammer et al., 2007; Ufelle & Barchowsky, 2019; Wayland & Scheuhammer, 2011). Egg metal concentrations reflect fairly well maternal concentrations (Ackerman et al., 2016), and transfer to eggs represents an important route of elimination from mother birds (Burger, 1994). However, only unhatched eggs can be collected from a protected and near threatened species, and their use introduces variability in contaminant concentrations due to differences in embryonic development, laying order and desiccation (see below). Unhatched eggs may also contain higher contaminant levels than successfully hatched eggs, potentially leading to overestimation of population exposure levels.

Our earlier work characterized temporal trends and spatial distribution of persistent halogenated aromatic hydrocarbons in a long-term series of unhatched osprey eggs (Viluksela et al., 2024). The present study focuses on metals using the same sample material. The metals include the toxic nonessential metals Hg, Cd, and Pb, and the essential metals with toxic potential Cu, Zn, Se, and Cr. In addition, three precious metals of the platinum group, i.e., platinum (Pt), palladium (Pd), and rhodium (Rh), were analyzed, but not detected.

The objective of this study was to analyze temporal changes in metal concentrations in osprey eggs from two Finnish environments: a marine area in the Gulf of Bothnia and an industrially influenced lake system. We hypothesize that (1) temporal trends will be metal‑specific with declines in Hg and Pb following reduced emissions and regulatory measures, and more variable declines for Cd and other metals linked to local sources; and (2) spatial differences will reflect sources and history of emissions together with levels in prey fish as indicated by data in the Accumulation register on pollutant concentrations of the Finnish Environment Institute (Finnish Environment Institute, Metadata Portal, 2025). By comparing these sites, we aim to distinguish regional patterns of exposure and to extend our earlier work on persistent halogenated aromatic hydrocarbons (Viluksela et al., 2024). Currently, no trend analyses exist that compare concentrations of toxicologically relevant metals between Baltic and limnic environments, particularly in a piscivorous apex predator species.

## Materials and methods

### Study areas

**Fig. 1:**
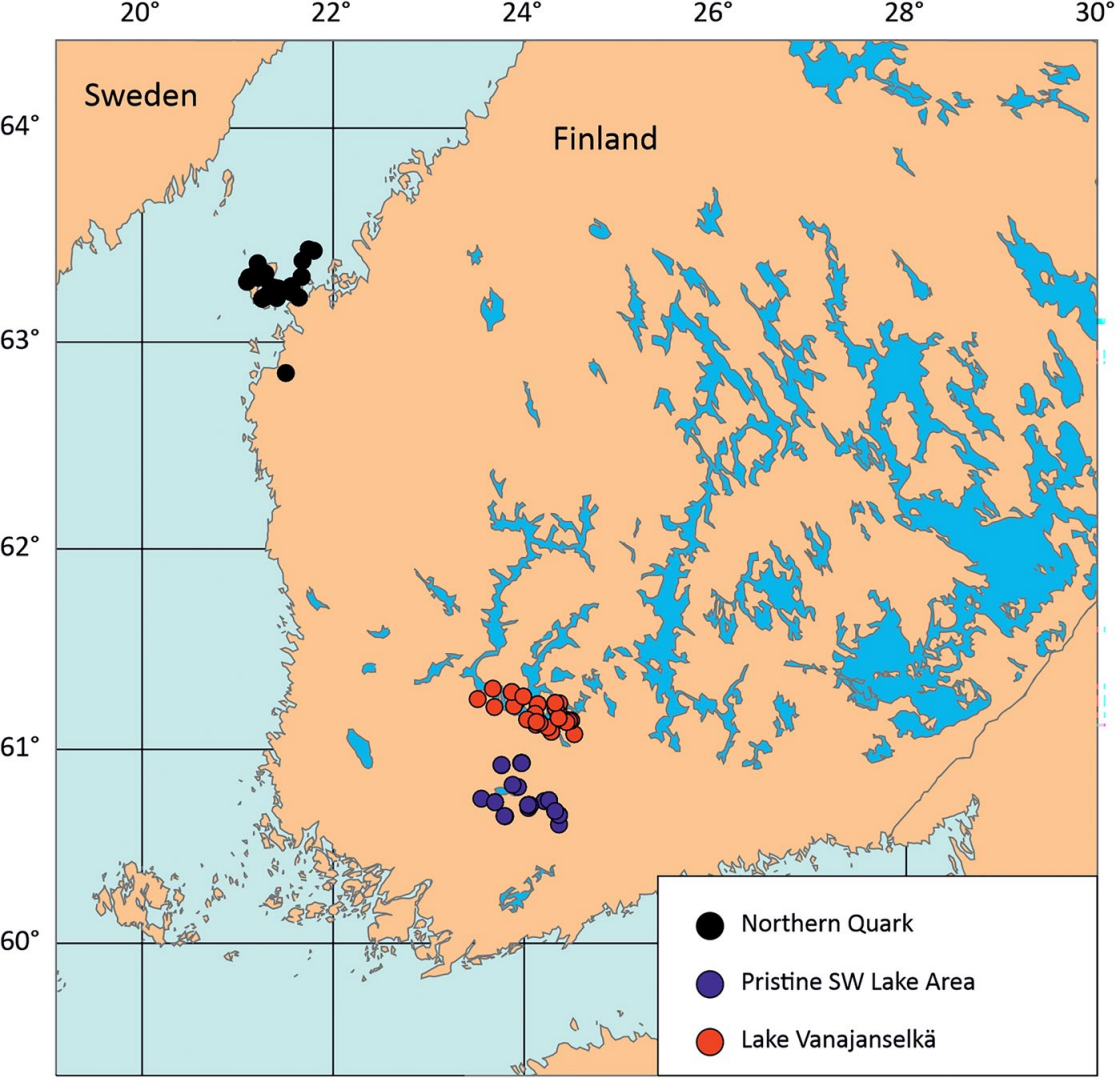
Map showing the study regions and sampling sites. The map was generated using the PIA mapping tool (AB) with coordinates sourced from the CIA public domain database

The study areas were Northern Quark, located in the Gulf of Bothnia (Baltic Sea) on the west coast of Finland, and Lake Vanajanselkä, a large eutrophic-dystrophic lake system in SW Finland, located about 280 km south-southeast from Northen Quark (Fig. 1). The Northern Quark Archipelago is affected by metal contamination from acid sulfate soils, mainly via the catchment of Kyrönjoki river and smaller rivers, which is reflected in increased levels of Mn, Zn, Co, Cu, Cd, and some other metals (Virtasalo et al., 2020). The levels increased during 1960–1970 due to intensive artificial drainage, and metal deposition has remained at high level since the 1980s. Lake Vanajanselkä has been polluted by large amounts of industrial effluents from wood-processing, fiber, metal, and food production industries and diffused sources from agriculture and forestry and municipal sewage since the 1870 s (Kansanen & Aho, 1981; Kansanen & Jaakkola, 1985; Rautalahti-Miettinen, 1977). In sediments, Zn, Cd, and Cu have exhibited distinct enrichment profiles, mainly due to Zn-rich wastewaters from a chemical fiber factory producing rayon staple fiber since 1943 with reported Zn emissions reaching 800 kg/day in 1972. The loading occurred during 1950–1980, with substantially reduced inputs thereafter. THg was also analyzed in samples from the Pristine SW Lake Area, a rural highland area of small lakes and peat bogs located about 45 km south of Lake Vanajanselkä.

### Collection of osprey eggs

A total number of 114 unhatched (addled) eggs were collected in 1972–2005, on average 3.4 eggs/year (Table 1) by participating licensed bird ringers when they were checking the occupied territories and ringing the nestlings in the nationwide monitoring Project Pandion led by the Ringing Centre of the Finnish Museum of Natural History and authorized by the Ministry of Environment (Viluksela et al., 2024). The eggs were collected within 4–8 weeks after the normal hatching time. A complete list of samples indicating the collection year, area, and results of analyses is shown in Online Resource 1. The eggs were weighed and measured; contents were stored in glass vials and frozen at −20°C for further analysis.

**Table 1:** Number of osprey eggs analyzed and the years during which they were collected in the different study areas

	Total Hg		Methyl Hg		Other metals	
	Number	Years	Number	Years	Number	Years
**Study area**						
Northern Quark	38	1979–2005 27	33	1979–2003	28	1979–2003
Lake Vanajanselkä	38	1972–2005 34	33	1972–2003	33	1972–2003
Pristine SW Lake Area	38	1972–2004 33				
Sum	114		66		61	

### Chemical analyses

THg and MeHg analyses were carried out at the IVL Swedish Environmental Research Institute Ltd. (Gothenburg, Sweden) and Cd, Cr, Cu, Pb, Se, Zn, Pd, Pt, and Rh were analyzed at the Finnish Environment Institute (Helsinki, Finland) using accredited methods. The IVL laboratory is accredited for analysis of THg and MeHg in water (SWEDAC no 1213) and the Finnish Environment Institute laboratory for analysis of Cd, Cr, Cu, and Pb in environmental samples (water, biological material, soil, sediment, and sludge) (FINAS no. T003).

Pretreatment of solid samples before THg analysis was performed through H_2_SO_4_/HNO_3_ digestion. THg analysis was performed after BrCl oxidation followed by SnCl_2_ reduction and precollection of volatilized Hg^0^ on gold traps followed by thermal desorption and cold vapor atomic fluorescence spectroscopy (CVAFS) detection.

MeHg was quantified after alkaline digestion, ethylation, and GC separation. Samples were digested in hot methanolic potassium hydrochloride (2–4 h), diluted with methanol, and ethylated using sodium tetraethylborate. This procedure converted MeHg to methylethylmercury and inorganic Hg(II) to diethylmercury. The volatile derivatives were purged with nitrogen, trapped on Tenax, thermally desorbed in helium, and separated by isothermal gas chromatography. The separated compounds were then pyrolyzed at 700–800 °C to elemental Hg and quantified by CVAFS, following the same detection protocol as for THg.

Detection limits for THg and MeHg were 0.06 ng/l, derived from 3 times the standard deviation of method blanks. Accuracy was monitored throughout the analyses with the certified reference materials DOLT-2 and DORM-2, respectively, giving typical recoveries of 97 ± 7% for MeHg and 100 ± 4% for THg.

Pretreatment of the samples before analysis of the other metals (Cd, Cr, Cu, Pb, Se, Zn, Pd, Pt, and Rh) was performed according to the US EPA Method 3051 A (Rev. 1, 1998). The samples were digested using a closed vessel microwave digestion system. The samples were weighed in TFM vessels and 5 ml concentrated HNO_3_ was added. The samples were heated with a microwave program consisting of several power steps with a duration of 20 min. After running the program and cooling the vessels, the samples were quantitatively transferred to acid leached polypropylene test tubes and diluted to 25 ml with deionized water. Before the elemental analysis, the samples were further diluted 10× with deionized water. Indium was added as an internal standard with a final concentration of 10 µg/l. The metals were determined by inductively coupled plasma mass spectrometry (ICP-MS) according to ISO 17294-2:2003 (2003). The reference material was DORM-2 (Dogfish Muscle Certified Reference Material for Trace Metals, National Research Council, Canada). Limits of detection (LOD), limits of quantification (LOQ), and typical recoveries are shown in Online Resource 2, Table S1.

Results are expressed in µg/g wet weight (ww). Values falling below LOQ were assigned a value of zero. For geometric mean calculations, values were substituted with half of the lowest quantified concentration when < 30% of samples from a given area were zero.

### Statistical analyses

The trend analyses were carried out using the PIA statistical application developed by Anders Bignert (Bignert, 2007) in three steps: (1) Log-linear regression analyses were carried out for the entire study period. The regression line indicates the annual percentage change and *r*^2^ is the coefficient of determination with a *p*-value for a two-sided test (H_0_: slope = 0). To avoid exaggerated influence of a single or a few data points at the end of the line, (2) the Mann-Kendall trend test (Gilbert, 1987; ICES, 1995) was carried out as a non-parametric alternative to the regression analysis resulting in Kendall’s tau (*τ*) and the corresponding *p*-value. (3) To identify non-linear trends a 5-point running mean smoother was applied and analysis of variance (ANOVA) was used to test if the smoother explains significantly more than (a) the overall mean concentration (a straight line) and (b) the log-linear regression line, considering the loss of degrees of freedom related to the smoother (Nicholson et al., 1998). To avoid potential leverage influence from extreme values on the regression analyses (due to e.g. desiccated egg content), values below 3.0× IQR of the lower quartile and values higher than 3.0 × IQR of the upper quartile, i.e., outside “Tukey’s outer fence” (Foreman, 2014; Tukey, 1977) have been detected and excluded.

Statistical comparisons of concentrations between study areas were performed using analysis of variance (ANOVA) for three‑area comparisons and two‑sample *t*‑tests for two‑area comparisons, conducted with Statistix 9.0 (Analytical Software, Tallahassee, FL, USA).

### Study limitations

Only unhatched osprey eggs could be used for contaminant analysis, which introduces several potential confounding factors including variability of the stage of embryonic development, unknown laying order, and possible desiccation (Bignert et al., 1995; Orłowski et al., 2016a, 2016b, 2017). Unhatched eggs may also contain inherently higher contaminant levels than successfully hatched eggs. Possibility for post-hatching microbiological degradation of samples can compromise analysis of non-persistent compounds (Herzke et al., 2002) but does not affect metal concentrations.

The study covers only two locations, which limits the generalizability of the findings to other marine and freshwater systems. The lack of simultaneous high-resolution data on environmental covariates (e.g., water and sediment chemistry) and composition of prey fish impedes the estimation of the contribution of local point sources. The last samples were collected in 2005, creating a temporal gap that reduces the ability to infer current exposure levels. However, the nesting success of the osprey population has gradually improved over the study period in line with decreasing contaminant levels and stabilized to the current rate since 2000 (see below), which provides a contextual support for the current relevance.

## Results

**Fig. 2:**
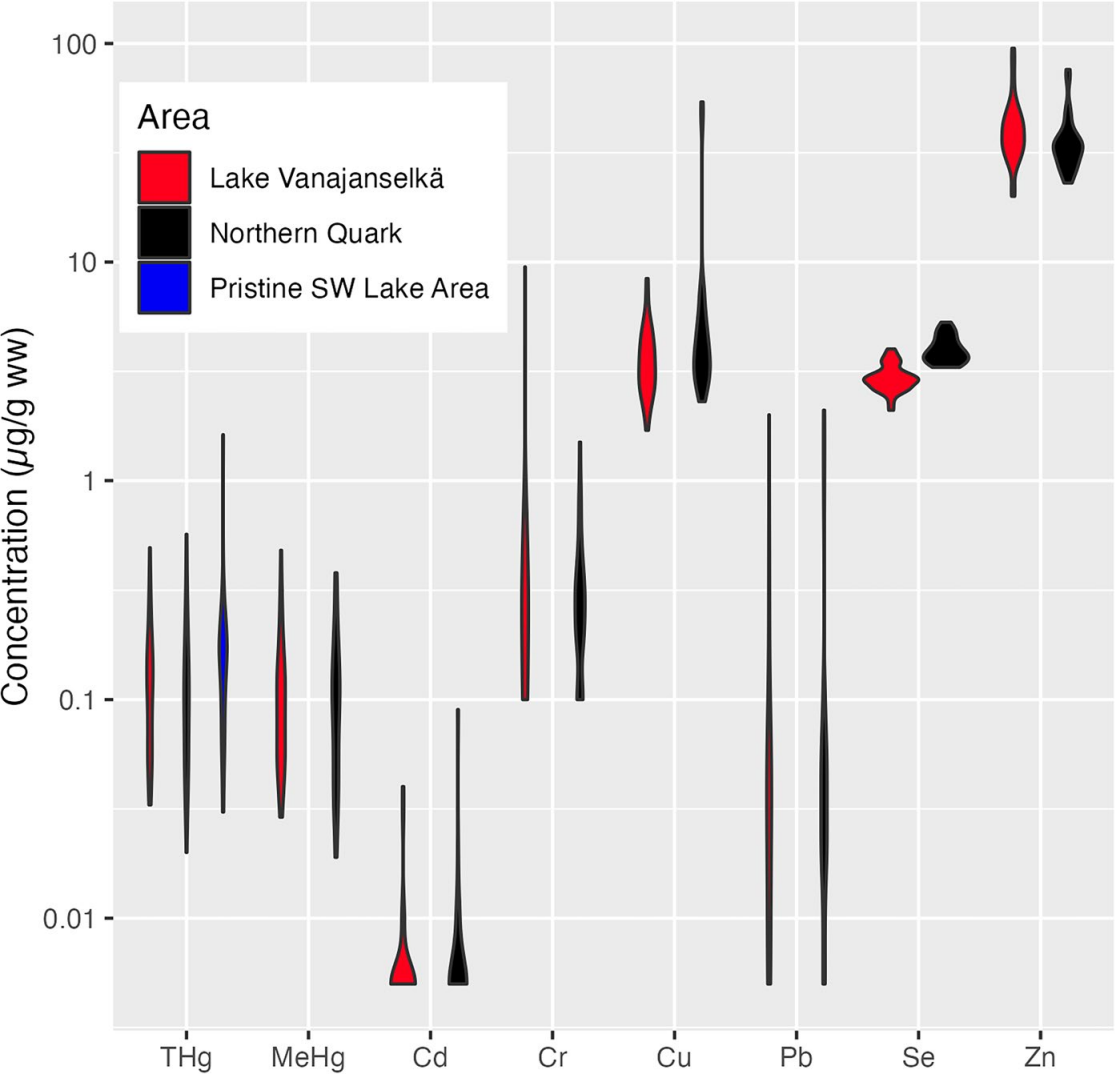
Metal concentration ranges in osprey eggs shown on a logarithmic scale. In the violin plots, the width represents the frequency of observations. Concentration ranges of the essential metals Se, Zn, and Cu were narrow, and Se concentration was significantly (*p* < 0.001, *t*-test) higher in the marine Northern Quark than in Lake Vanajanselkä

The concentration ranges of all metals quantified in osprey eggs and the frequency of observations are shown in Fig. 2 and in Tables 2 and 3. Concentration ranges of the essential metals Se, Zn, and Cu (especially in Lake Vanajanselkä) were narrow as compared to the other metals. Concentrations of Pt, Pd, and Rh in all samples were below LOQ, < 0.01 mg/kg for Pt and Rh, and < 0.02 mg/kg for Pd. Trend analyses revealed significant declining trends for Pb, Cr, MeHg, and THg, and their decline was marginally faster in Northern Quark than in Lake Vanajanselkä (see below). Trends for the essential metals Se, Cu, and Zn were nonsignificant. Metal concentrations did not significantly differ between the study areas, except for Se, which was significantly higher in Northern Quark than in Lake Vanajanselkä.

**Table 2:** Comparison of THg, MeHg, and Se concentrations in eggs from this study with values reported in other studies on ospreys, sea eagles, or other piscivorous avian species considered relevant in terms of trophic level, location, or time frame (µg/g ww, geometric mean and range, unless otherwise stated)

Species	Country, area	Year	*n*	THg	MeHg	Se	Reference
Osprey	Finland, all areas	1972–2005 MeHg, Se: 1972–2003	114	0.111 0.020–1.622	0.089 0.019–0.480	3.37 2.10–5.30	This study
Osprey	Finland, Northern Quark	1979–2005 MeHg, Se: 1979–2003	38	0.096 0.020–0.568	0.087 0.019–0.380	3.96 3.30–5.30	This study
Osprey	Finland, Lake Vanajanselkä	1972–2005 MeHg, Se: 1972–2003	38	0.106 0.033–0.493	0.091 0.029–0.480	2.94 2.10–4.00	This study
Osprey	Finland, Pristine SW Lake Area	1972–2004	38	0.136 0.031–1.622			This study
Osprey	Finland, Northern Quark	1971–1972	6	0.11^a^ 0.04–0.24^a^			Koivusaari et al., 1972
Osprey	SW Finland	1981–1982	11	0.18 0.1–0.4			Häkkinen & Häsänen, 1980
Osprey	USA, Maryland	1973	10	0.07 0.02–0.13			Wiemeyer et al., 1988
	USA, Florida, Everglades	1973	5	0.070.04–020			
	USA, New Jersey	1978	9	0.05–0.25			
Osprey	Canada, Northern Québec	1989–1991	51	0.20^b^ 0.06–0.54			DesGranges et al., 1998
Osprey	USA, New Jersey, Delaware Bay area	1998	17	0.12 0.04–0.26			Clark et al., 2001
Osprey	Mediterranean: Balearic Corsica Tuscany	2005–2017 2010–2012 2012–2018	10 7 7	0.20 ± 0.16^c^ 0.20 ± 0.17 0.10 ± 0.07		0.35 ± 0.12^c^ 0.38 ± 0.15 0.30 ± 0.16	Monti et al., 2020
White-tailed sea eagle	Sweden, Baltic coast	1965–1978	75	0.58^b^ 0.17–1.14			Helander et al., 1982
White-tailed sea eagle	Sweden, Lapland	1965–1978	4	0.28^b^ 0.19–0.37			Helander et al., 1982
Guillemot	Sweden, Baltic, Stora Karlsö	2006	255	0.26 0.23–0.31^d^			Bignert et al., 2010
Raptors, seabirds, other fish-eating birds	Multiple locations	Published 1994–2000	Hg: 68^d^ Se: 13	0.340^e^ 0.07–7.290		1.100^e^ 0.300–7.100	Burger, 2002

^a^Estimated from a plot

^b^Mean

^c^Mean ± SD

^d^Number of studies

^e^Median, range

**Table 3:** Comparison of Cd, Cr, Cu, Pb, and Zn concentrations in eggs from this study with values reported in other studies on ospreys, sea eagles, or other piscivorous avian species considered relevant in terms of trophic level, location, or time frame (µg/g ww, geometric mean and range, unless otherwise stated)

Species	Country, area	Year	*n*	Cd	Pb	Cr	Cu	Zn	Reference
Osprey	Finland, all areas	1972–2003	61	0.004^a^ ND^b^−0.09	0.036 ND-2.10	0.276 ND-9.50	3.97 1.70–54.00	37.2 20.0–95.0	This study
Osprey	Finland, Northern Quark	1979–2003	28	0.005^a^ ND-0.09	0.043 ND-2.10	0.267 ND-1.50	4.67 2.30–54.00	34.6 23.0–76.0	This study
Osprey	Finland, Lake Vanajanselkä	1972–2003	33	0.003^a^ ND-0.04	0.030 ND-2.00	0.288 ND-9.50	3.45 1.70–8.40	39.4 20.0–95.0	This study
Osprey	USA, New Jersey, Delaware Bay area	1998	17		0.30 0.20–0.68				Clark et al., 2001
Osprey	Mediterranean: Balearic Corsica Tuscany	2005–2017 2010–2012 2012–2018	10 7 7	0.0005 ± 0.0002^c^ 0.0006 ± 0.0002 0.0008 ± 0.0005	0.011 ± 0.011^c^ 0.024 ± 0.010 0.005 ± 0.006		0.56 ± 0.10^c^ 0.52 ± 0.21 0.52 ± 0.27	5.65 ± 1.34^c^ 4.10 ± 1.71 4.77 ± 1.72	Monti et al., 2020
Guillemot	Sweden, Baltic, Stora Karlsö	2003	80	0.0002 0.0002–0.0004^d^	0.006 0.005–0.008^d^	0.0250 0.0202–0.0312^d^	0.60 0.56–0.67^d^	8.94 7.90–9.78^d^	Bignert et al., 2010
Raptors, seabirds, other fish-eating birds	Multiple locations	Published 1994–2000	Cd:32^e^ Pb: 29 Cr: 21	0.015^f^ 0.002–0.600	0.190^f^ 0.020–6.700	0.210^f^ 0.010–1.000			Burger, 2002

^a^Mean

^b^*ND*, non-detectable

^c^Median ± SD

^d^95% confidence interval

^e^Number of studies

^f^Median, range

### THg and MeHg

**Fig. 3:**
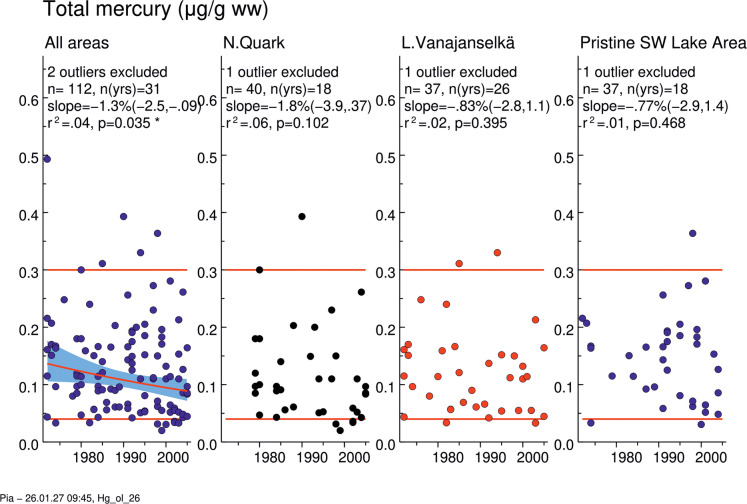
Temporal trends of THg in osprey eggs in the whole dataset and in different study areas. Circles indicate individual data, and a red regression line is shown if *p* < 0.05 (two-sided regression analysis). There was a significant decrease in THg and MeHg in all areas (annual decrease 1.30%). The shaded area is the 95% confidence band for the regression line and red horizontal lines indicate the toxicity reference values for low (0.04 µg/g ww) and moderate (0.3 µg/g ww) according to Ackerman et al., 2024. Abbreviations: *n*, total *n* of observations; *n*(yrs), total *n* of years; slope, annual change (%); *r*^*2*^, coefficient of determination; *p*-value for a two-sided test (H0: slope = 0)

THg and MeHg concentrations of the whole study period did not differ among study areas (Table 2, Fig. S2). There was a significant decreasing trend in all study areas combined for both THg and MeHg (annual decrease 1.30 and 1.93%, respectively) (Figs. 3, S1, Table S2). Otherwise, the decreasing trends in the study areas were nonsignificant, but suggested faster decrease in Northern Quark than in Lake Vanajanselkä and in Pristine SW Lake Area. On average, MeHg contributed 87.5% of THg in all areas, 91.6% in Northern Quark and 83.4% in Lake Vanajanselkä (Fig. S3). Correlation between MeHg and THg concentration was strong both in Northern Quark (*R*^2^ = 0.91) and in Lake Vanajanselkä (*R*^2^ = 0.88) (Fig. S4).

### Se

Se concentrations of the whole study period were significantly higher (*p* < 0.001, *t*-test) in Northern Quark (mean 4.00, median 3.80 µg/g ww) than in Lake Vanajanselkä (mean 2.96, median 2.90 µg/g ww) (Table 3, Fig. S5). The trends were nonsignificant in the whole dataset and in both study areas (Fig. S6, Tables 2S2). There was no correlation between Se and THg concentrations (Fig. S7).

### Cd

Cd concentrations in all samples were very low, and in 50 samples out of 61, the levels were below LOQ, and Cd was not quantified after 1985 in Northern Quark and after 1997 in Lake Vanajanselkä (Table 3, Fig. S8). Because only 6 samples from Northern Quark and 5 from Lake Vanajanselkä were quantified, the dataset was considered too limited for a reliable trend analysis.

### Pb

**Fig. 4:**
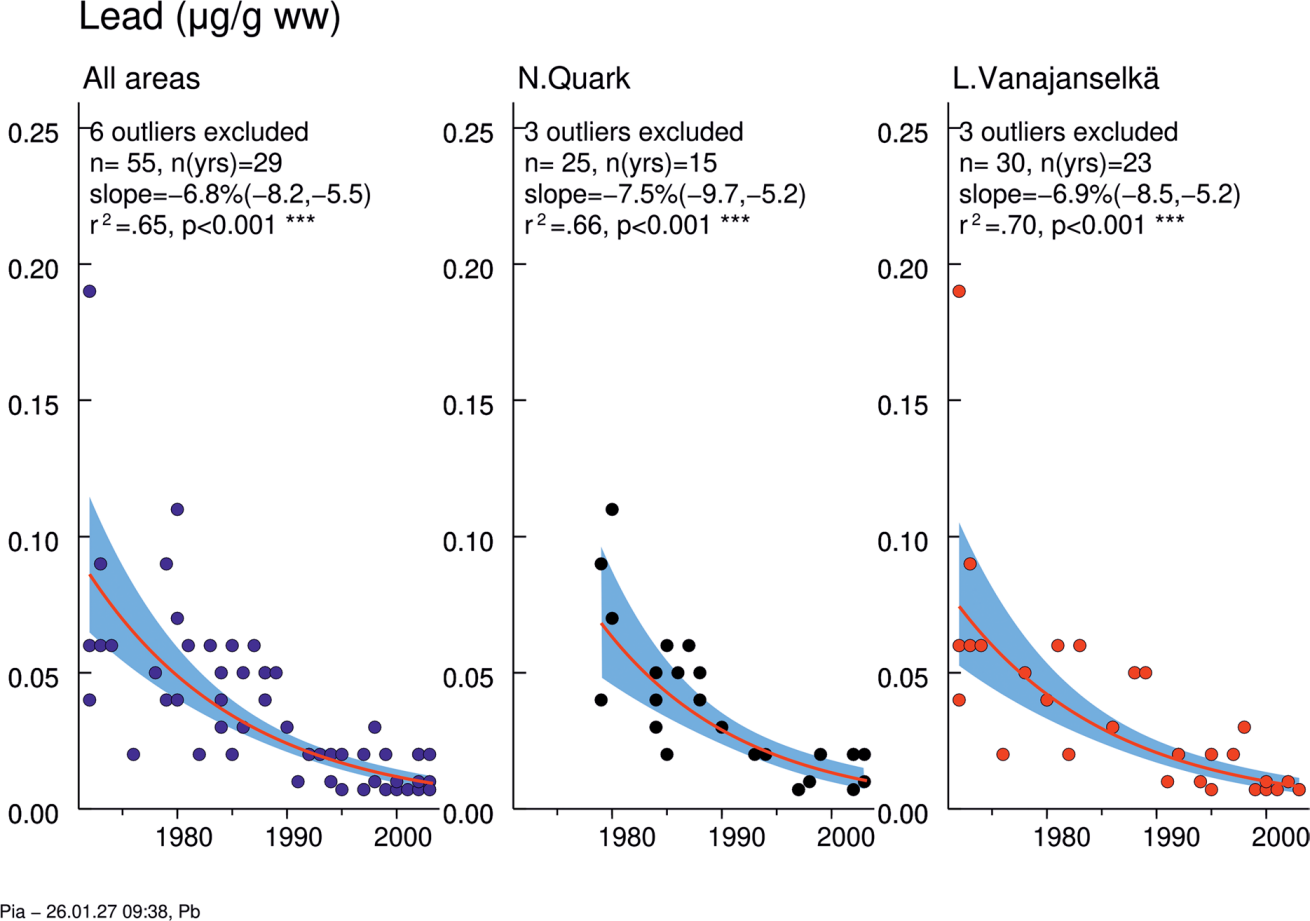
Temporal trend of Pb levels in osprey eggs in different study areas. Blue circles indicate individual data, and a red regression line is shown if *p* < 0.05 (two-sided regression analysis). Pb concentrations decreased significantly in the whole dataset (annual decrease 6.85%) and in both study areas (annual decrease 7.49 and 6.87%, respectively). The shaded area is the 95% confidence band for the regression line. Abbreviations: *n*, total *n* of observations; *n*(yrs), total *n* of years; slope, annual change (%); *r*^*2*^, coefficient of determination; *p*-value for a two-sided test (H0: slope = 0)

No significant differences in Pb concentrations between study areas were found (Table 3; Fig. S9). In 1979, there were three samples with high Pb levels from Northern Quark (the two highest of them had also the highest Cu levels) and in 1985 two samples from Lake Vanajanselkä (the same samples had the highest Cr levels, and the highest one had the highest Zn level). These are biological outliers, because the eggs were not desiccated. A significant decreasing trend was found in the whole dataset and in both study areas (annual decreases 6.87, 7.49, and 6.87%, respectively) (Fig. 4, Table S2). The decrease was faster in Northern Quark than in Lake Vanajanselkä.

### Cr

**Fig. 5:**
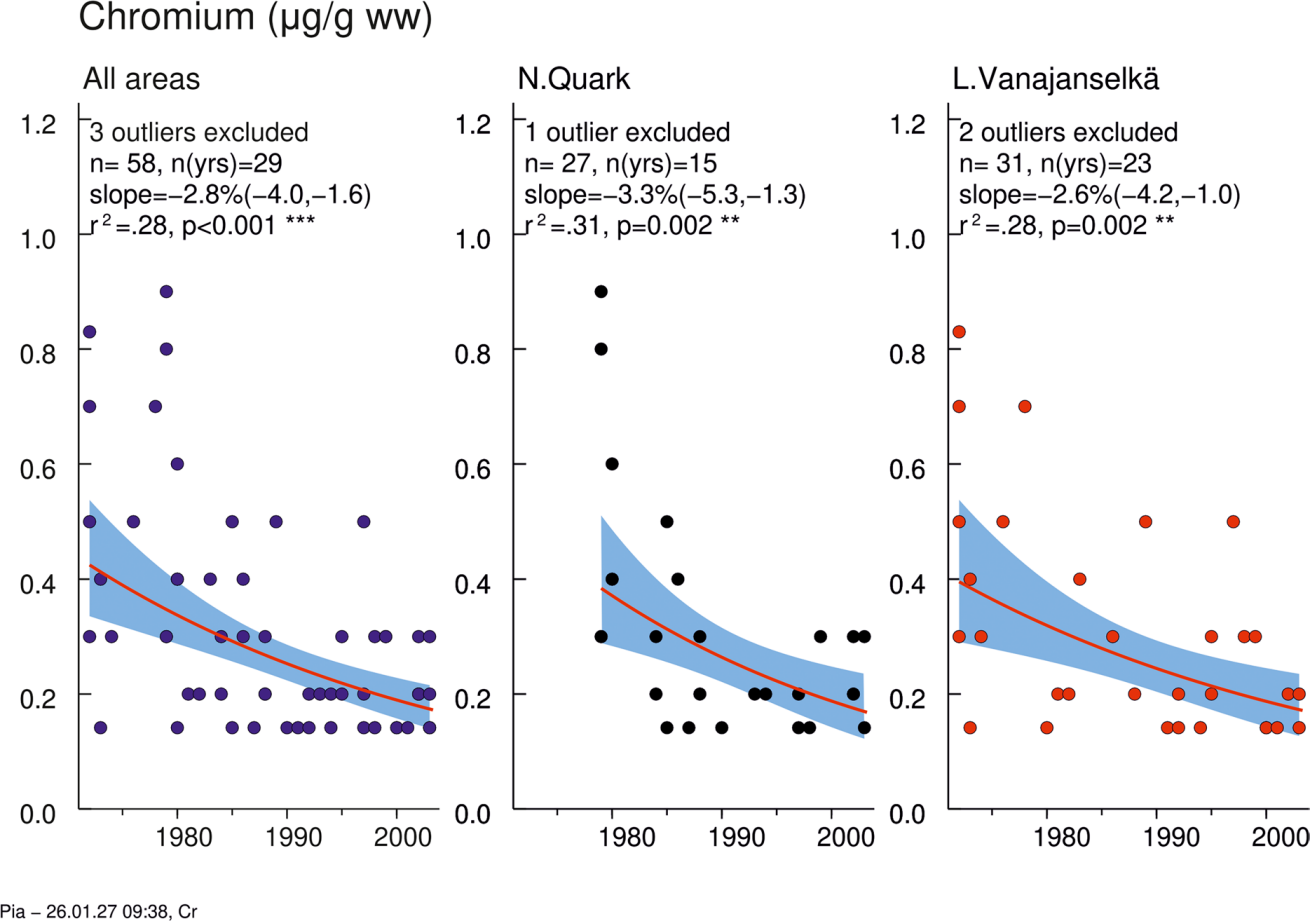
Temporal trend of Cr in osprey eggs in different study areas. Blue circles indicate individual data, and a red regression line is shown if *p* < 0.05 (two-sided regression analysis). Cr concentrations decreased significantly in the whole dataset (annual decrease 2.75%) and in both study areas (annual decrease 3.25 and 2.55%, respectively). The shaded area is the 95% confidence band for the regression line. Abbreviations: *n*, total *n* of observations; *n*(yrs), total *n* of years; slope, annual change (%); *r*^*2*^, coefficient of determination; *p*-value for a two-sided test (H0: slope = 0)

Cr concentrations of the whole study period were not significantly different in Northern Quark and in Lake Vanajanselkä (Table 3, Fig. S10). The two highest concentrations were from Lake Vanajanselkä (1985), and they had also the highest Pb and Cu levels. There was a statistically significant decreasing trend in the whole dataset and in both study areas (annual decreases 2.84, 3.35, and 2.63%, respectively) (Fig. 5, Table S2). The decrease was faster in Northern Quark than in Lake Vanajanselkä.

### Cu

There were no significant differences in Cu concentrations between study areas (Table 3, Fig. S11). The two highest values were from Northern Quark (1979), the same samples with high Pb levels. No temporal trends were detected in the whole dataset and in both study areas (Fig. S12, Table S2).

### Zn

Zn concentrations of the whole study period were not significantly higher in Lake Vanajanselkä than in Northern Quark (Table 3, Fig. S13). The highest concentration was in Lake Vanajanselkä (1985), the same sample with the highest Pb, Cr, and Cu levels. The trends were nonsignificant in the whole dataset and in both study areas (Fig. S14, Table S2).

## Discussion

### Temporal trends

This study found significantly decreasing trends for Pb, Cr, MeHg, and THg in osprey eggs, with the decline marginally faster in Northern Quark. Pb and Hg are toxic, nonessential metals, whereas Cr is essential but can be toxic at elevated concentrations. The decreasing trends closely matched reductions in atmospheric deposition measured by moss biomonitoring in Finland (Poikolainen et al., 2004), and the Pb and THg trends were comparable to those reported for guillemot eggs and tawny owl feather in Nordic countries (Bignert et al., 2010; Bustnes et al., 2022). The decrease was also similar to that observed for persistent halogenated aromatic hydrocarbons in the same samples (Viluksela et al., 2024). In contrast, Se, Cu, and Zn showed no significant temporal trends, concentration ranges remaining relatively narrow and stable, likely because these metals are physiologically regulated (Ufelle & Barchowsky, 2019). Cr was the only essential metal with a significant decline in our dataset; this contrasts with guillemot and tawny owl studies that found no trend. This difference may reflect higher Cr concentrations in our samples compared to those in samples from other locations (Tables 2 and 3).

### Spatial differences

Despite the pollution history of Lake Vanajanselkä and the riverine input of metals from acid sulfate soils to Northern Quark Archipelago, the levels were not exceptionally high, and metal concentrations in osprey eggs did not differ between the study areas (except for Se; see below). Data on THg, Pb, Cu, and Zn in prey fish from the Accumulation register (Finnish Environment Institute, 2025) likewise showed no spatial differences. Together these observations suggest that long-range atmospheric deposition, rather than local sources, was the dominant determinant of egg metal concentrations during the study period. This pattern contrasts with the sum concentrations of persistent halogenated aromatic hydrocarbons in the same samples, which were markedly higher in Lake Vanajanselkä than in the other areas (Viluksela et al., 2024). Contrary to the other metals, Se concentrations were significantly higher in Northern Quark than in Lake Vanajanselkä, consistent with generally higher Se levels in marine compared with freshwater environments (Amouroux et al., 2001; Ferraz et al., 2023).

### THg and MeHg

THg and MeHg concentrations decreased significantly in all study areas combined (1.3% and 1.9%, respectively). The annual decrease of THg was faster in body feathers of white-tailed sea eagles collected from the Central Swedish Baltic Sea coast in 1967–2011 (Sun et al., 2019), and the decrease was more pronounced (3.8%) between 1967 and 1985 than between 1985 and 2011 (2.2%). The higher Hg concentrations and faster decrease in Sweden compared to Finland reflect the more intensive industrial and agricultural Hg use in the past (Munthe et al., 2007). In guillemot eggs from Stora Karlsö, southern Baltic Proper, collected in 1969–2006, the annual THg decrease was 1.5% (Bignert et al., 2010). In an extensive database of over > 54,000 fish entries from > 3000 lakes from boreal and subarctic Fennoscandia (1965–2017), the annual decrease of THg was slower than in osprey eggs, 0.3% for pike and 0.7% for perch in the boreal region (Braaten et al., 2019). However, the annual decrease of atmospheric Hg deposition was 1.8% in Finland between 1995 and 2000 (Poikolainen et al., 2004) and 1.1% in Europe between 1995 and 2005 (Harmens et al., 2010) closely reflecting the rate of decrease observed in osprey eggs.

Total mercury concentrations in osprey eggs of the present study were in the same range with those in osprey eggs from Northern Quark (1971–1972) (Koivusaari et al., 1972), SW Finland (1981–1982) (Häkkinen & Häsänen, 1980), Northern Québec, Canada (19,891,991) (DesGranges et al., 1998), and New Jersey, USA (1978) (Clark et al., 2001), and only slightly lower than the more recent concentrations in osprey eggs from the Mediterranean (2005–2018) (Monti et al., 2020). Our THg concentrations were lower than those in white-tailed sea eagle eggs from Swedish Baltic Coast and Lapland (1965–1978) (Helander et al., 1982), and in guillemot eggs from Stora Karlsö (2006) (Bignert et al., 2010). For comparison, a synthesis of 68 studies published between 1994 and 2000 on eggs of raptors, seabirds, and other fish-eating birds mainly from North America (Burger, 2002) revealed predominantly higher THg levels than those in the present study. Aligned with previous data (Ackerman et al., 2013), most (87.5%) of THg in our database was MeHg.

In line with similar Hg levels in osprey eggs across our study areas, the THg concentration data in the Accumulation register on pollutant concentrations (Finnish Environment Institute, 2025) indicate that the levels in pike muscle from Northern Quark (1970–1992, median 0.21, range 0.08–1.22 µg/g ww, *n* = 40) and Lake Vanajanselkä (1971–2002, median 0.18, range 0.05–0.39 µg/g ww, *n* = 30) were quite similar.

Do the egg Hg concentrations measured reach levels likely to cause toxic effects in offspring? Using MeHg toxicity reference values from Ackerman et al. (2024), 7.0% of our samples were below the threshold for low injury (0.04 µg/g ww), 86.0% fell within low injury range (0.04 to < 0.3 µg/g ww), 6.1% were within moderate injury range (0.3 to < 0.7 µg/g ww), and a single sample fell in high injury range (0.7 to < 1.8 µg/g ww) (Fig. 3, Online Resource 1). All samples collected after 1998 were at or below the low injury threshold. On this basis, Hg, alone or together with dioxin-like compounds, could have contributed to reduced reproductive success before 2000, as observed in Lake Vanajanselkä (Viluksela et al., 2024). The high Se to Hg ratio we observed may have offered protection against Hg-induced reproductive toxicity (see below; Adams & Duguay, 2025). A mixture effect between Hg and Pb is also plausible, given the reported synergistic interaction between these metals in raising feather corticosterone in nestling red kites (*Milvus milvus*) (Powolny et al., 2020).

### Se

Se concentrations were significantly higher in osprey eggs from Northern Quark than from Lake Vanajanselkä, and there was no temporal trend. In line with our data, the lack of temporal trend was also reported in guillemot eggs from Baltic Proper (2009–2016) (Bignert et al., 2017) as well as in tail feathers of osprey nestlings from a lake in southern Sweden (1969–1998) (Odsjö et al., 2004) and in tail feathers of tawny owl (*Strix aluco*) in central Norway (1986–2005) (Bustnes et al., 2022). Se concentrations in Finnish lake waters are slightly lower than in other Nordic countries, Se fortification of fertilizers has not increased Se sedimentation in Finnish lakes (Wang et al., 1995), and no increase was observed in Se concentrations of osprey eggs after initiation of fortification in 1985. Average Se concentration (± SD) in perch muscle from 12 Finnish lakes was 0.33 ± 0.26 µg/g ww (using a moisture content of 80% (Florek & Staszowska, 2013)), which is similar to or lower than in perch muscle from Sweden and North America. In the compilation of 13 studies on eggs of raptors, seabirds, and other fish-eating birds, the median Se concentration was higher than that in the present study, i.e., 1.10 µg/g ww and range up to 7.10 µg/g ww (Burger, 2002).

Se has been shown to mitigate toxicity of Hg through the action of Se-dependent enzymes that protect cells against oxidative damage. Generally, molar Se to Hg ratios > 1 are protective against Hg toxicity (Adams & Duguay, 2025). In this study, the Se to Hg ratios calculated from GM concentrations indicate Se was in large excess: 41 in Northern Quark and 28 in Lake Vanajanselkä. The ratios increased over time, rising from 36 and 22 in samples collected before 1981 to 65 and 32 in samples collected since 2000, respectively. These ratios are higher than those in osprey eggs collected from the Mediterranean in 2005–2018 (1.8–3) (Monti et al., 2020).

### Cd

Cd concentrations were very low in both study areas and reflected the low levels in prey fish (Finnish Environment Institute, 2025). Mean Cd concentrations of our samples were about 6 times higher than reported for osprey eggs from the Mediterranean (2005–2018) (Monti et al., 2020), and about 20 times higher than guillemot eggs from southern Baltic Proper (2003) (Bignert et al., 2010), and nearly 4 times lower than the median from the compilation of 32 studies on eggs of raptors, seabirds and other fish-eating birds (Burger, 2002). Although species-specific toxicity thresholds for developing embryos are not well established, adverse effects in predatory birds have been reported at 1 µg/g ww and above (cf. Burger & Elbin, 2015). Even the highest Cd level of 0.09 µg/g ww observed in this study was an order of magnitude below this threshold, indicating no evidence of toxicological concern.

### Pb

Pb concentrations were similar in both study areas and showed a significant and consistent decline across the entire dataset (annual decrease 6.9%), and in Northern Quark and Lake Vanajanselkä (annual decrease 7.5% and 6.9%, respectively). An annual decrease of 13% between 1996 and 2003 was reported in guillemot eggs from southern Baltic Proper (Bignert et al., 2010). Consistent with our findings, the annual decreases were 4.7% in tail feathers of tawny owls from central Norway (1986–2005) (Bustnes et al., 2022), 4.9% in moss analyses in Finland during 1985–2000 (Poikolainen et al., 2004), and 4.5% in Europe during 1990–2005 (Harmens et al., 2010). The primary driver of these reductions in biota is the phase-out and ban of leaded gasoline, initiated in the Nordic countries during the 1980 s and completed by the mid-1990s.

Pb concentrations of the present study were up to 10 times lower than those in osprey eggs from Delaware Bay area, USA (1998) (Clark et al., 2001), but all our samples (except one) collected after 1990 were ≤ 0.02 µg/g ww, which is similar to osprey eggs from Mediterranean (2005–2018) (Monti et al., 2020). Our late samples were also only slightly above guillemot eggs from southern Baltic Proper (2003) (Bignert et al., 2010). Pb concentrations in prey fish were below LOQ (0.01 mg/kg) in Northen Quark (1979) and median concentrations were 0.04–0.05 mg/kg ww in Lake Vanajanselkä (1975) (Finnish Environment Institute, 2025).

No data is available for an accurate estimate of toxicity threshold of Pb in osprey eggs. However, a tentative estimate can be inferred based on an experimental study in hens exposed to Pb nitrate during a 20-day laying period, where Pb concentration in egg contents linked with significantly decreased laying rate was approximately 1.2 µg/g ww (Wang et al., 2021). Given that avian species may exhibit more sensitive endpoints of Pb toxicity, such as neurodevelopmental and neurobehavioral disturbances (Burger & Gochfeld, 2000), it is plausible that adverse effects could have occurred at Pb levels observed in this study, particularly among a few outliers in 1979–1985.

### Cr

Cr concentrations were similar between the study areas and showed a significant decline across the entire dataset and in both study areas, slightly faster in Northern Quark than in Lake Vanajanselkä (annual decreases 2.8%, 3.4%, and 2.6%, respectively). In guillemot eggs from southern Baltic Proper, the annual decrease between 1995 and 2003 was nonsignificant (Bignert et al., 2010). Similarly, no significant trend was observed in tawny owl tail feathers from central Norway (1986–2005) (Bustnes et al., 2022), although moss analysis in southern Norway showed an annual decrease of 2.8%. Further moss analyses indicated an annual decrease of 1.11% in atmospheric Cr deposition in Finland during 1985–2000 (Poikolainen et al., 2004) and 0.13% in Europe during 1990–2005 (Harmens et al., 2010).

Cr concentrations of the present study were markedly higher than those reported in more recent (2003) guillemot eggs (Bignert et al., 2010). However, the compilation of 21 studies on eggs of raptors, seabirds, and other fish-eating birds showed median Cr concentrations 24% below those reported here (Burger, 2002). Despite higher Cr levels in this study, the average median atmospheric Cr deposition was remarkably consistent across Finland, Norway, and Europe (Harmens et al., 2010; Poikolainen et al., 2004; Steinnes et al., 2011).

### Cu

Cu concentrations did not differ between the two study areas, and no significant trends were observed in the whole dataset or within either region. Similarly, nonsignificant trends were reported in guillemot eggs from southern Baltic Proper (1996–2016) (Bignert et al., 2017) and in tawny owl feathers from central Norway (1986–2005) (Bustnes et al., 2022). In contrast, moss analyses showed an annual decrease of 2.0% in atmospheric Cu deposition in Finland during 1985–2000 (Poikolainen et al., 2004) and 1.3% across Europe during 1990–2005 (Harmens et al., 2010).

Median Cu concentrations of our samples were 6.2–9.0 times higher than those in Mediterranean osprey eggs (2005–2018) (Monti et al., 2020). Data from the Accumulation register (Finnish Environment Institute, 2025) indicate comparable levels in pike muscle from Northern Quark (1979, range 0.11–0.20 µg/g ww, *n* = 3) and Lake Vanajanselkä (1975, range 0.21–0.23 µg/g ww, *n* = 3).

### Zn

Zn concentrations did not differ significantly between Lake Vanajanselkä and Northern Quark, and no significant trends were observed in the whole dataset or within either study areas. In guillemot eggs from southern Baltic Proper, an annual decrease of 2.8% was reported for 1996–2003 (Bignert et al., 2010). Similarly, no consistent trend for Zn was observed in tawny owl feathers from central Norway (1986–2005) (Bustnes et al., 2022). Moss analyses revealed an annual decrease of 1.6% in atmospheric Zn deposition in Finland during 1985–2000, primarily between 1995 and 2000 (Poikolainen et al., 2004), and 1.8% across Europe during 1990–2005 (Harmens et al., 2010).

Data from the Accumulation register (Finnish Environment Institute, 2025) indicate slightly higher levels in pike muscle from Lake Vanajanselkä (1975, range 3.20–4.20 µg/g ww, *n*=3) compared to Northern Quark (1979, range 3.30–3.50 µg/g ww, *n*=3), consistent with the minor difference in osprey eggs. Thus, the historical impact of Zn-rich wastewaters in Lake Vanajanselkä (Kansanen & Aho, 1981; Rautalahti-Miettinen, 1977) is only marginally reflected in levels in osprey eggs.

### Recovery of osprey population

A small number of eggs with highest THg and Pb concentrations exceeded toxicological thresholds and may have contributed to reduced reproductive success, together with the dioxin-like compounds previously documented in Lake Vanajanselkä (Viluksela et al., 2024). With the declining concentrations of these contaminants, the Finnish osprey population began to recover after the early 1970 s, increasing by 57% between 1975 and 1995 and by additional 27% between 1995 and 2019. According to the outcome of the nationwide monitoring program Project Pandion, the average productivity (chicks per active nest) of the population increased from 1.80 ± 0.02 (mean ± SE) in the 1970 s to 1.95 ± 0.04 in the 1980 s, 2.03 ± 0.02 in the 1990 s and stabilized at 2.00 ± 0.02 after 2000 (Saurola, 2021, 2025; Online Resource 2 Fig. S15).

## Conclusions

Pb, Cr, MeHg, and THg concentrations in osprey eggs declined significantly, consistent with reductions in atmospheric deposition, with the steepest decline in Pb following the phase-out of leaded gasoline. Only the highest THg and Pb concentrations reached potentially toxic thresholds and may have contributed to reduced reproductive success, alone or together with dioxin-like compounds. Metal concentrations in eggs showed no significant regional differences and mirrored concentrations in prey fish, highlighting the role of long-range atmospheric transport.

Over the study period, the Finnish osprey population recovered gradually, paralleling declines in both metals and persistent halogenated aromatic compounds. Although the use of unhatched eggs introduces variability related to embryonic stage, laying order, viability, and desiccation, the overall patterns remain robust.

Despite these limitations, the study reinforces the value of osprey as a sentinel species and highlights the importance of archived environmental specimen collections from apex predators for monitoring both legacy and emerging environmental contaminants. More systematic use of existing specimen banks, together with continued archiving of well-documented samples and associated metadata, including biological effect metrics, will improve our ability to identify chemicals of emerging concern and support more proactive risk management. When applied to emerging contaminants, this approach can serve as an early‑warning system, enabling retrospective screening for substances such as novel flame retardants, pharmaceuticals, and biocides, and guiding timely adjustments in regulatory and monitoring responses.

## Supplementary Information

Below is the link to the electronic supplementary material.

ESM 1 (XLSX. 25.2 KB)

ESM 2 (PDF.1.39 MB)

## Data Availability

All data generated or analyzed during this study are included in this published article and its supplementary information files.
