# Supplementary material for: Temporal trends and spatial differences of trace elements in osprey eggs in Finland
Source: Environ Monit Assess. 2026 May 23;198(6):643. doi: 10.1007/s10661-026-15460-5 (PMC13198479; doi:10.1007/s10661-026-15460-5)
Supplement: Supplementary file 2 — (PDF.1.39 MB) [file 10661_2026_15460_MOESM2_ESM.pdf]

Supplementary Information

Online Resource 2. Supplementary Tables and Figures

Environmental Monitoring and Assessment

Temporal trends and spatial differences of trace elements in osprey eggs in Finland

Matti Viluksela, Pertti Saurola, Juhani Koivusaari, Matts Finnlund, Timo Sara-Aho, Anders Bignert, Jouni T. Tuomisto, Hannu Kiviranta and Matti Verta

Corresponding author: Matti Viluksela, School of Pharmacy and Department of Environmental and Biological Sciences, University of Eastern Finland, Kuopio, Finland; Finnish Institute for Health and Welfare (THL) / Department of Public Health, P.O. Box 95, FI-70701 Kuopio, Finland, matti.viluksela@uef.fi

Online Resource Table S1 Limits of detection (LOD), limits of quantification (LOQ), and typical recoveries of the analytical methods

Metal	Limit of detection (mg/kg)	Limit of quantification (mg/kg)	Typical recovery % (mean±SD)
Cadmium (Cd)	0.003	0.01	114±8
Chromium (Cr)	0.07	0.2	90±6
Copper (Cu)	0.03	0.1	94±4
Lead (Pb)	0.003	0.01	90±8
Palladium (Pd)	0.007	0.02	
Platinum (Pt)	0.003	0.01	
Rhodium (Rh)	0.003	0.01	
Selenium (Se)	0.1	0.3	104±6
Zinc (Zn)	0.3	1.0	100±4
Total mercury (THg)	0.06[a]		100±4
Methylmercury (MeHg)	0.06[a]		97±7

[a] ng/l

Online Resource Table S2 Average annual change, 95% confidence interval and significance for metal concentrations in different study areas. Sampling years 1972-2005 for THg and 1972-2003 for the other metals. Outliers (0-6) were excluded as shown in Figs. 3-9. Statistical significance based on log-linear regression analysis, non-parametric Mann-Kendall trend test (Kendall's tau) and 5-point running mean smoother (ANOVA) for non-linear trends. Statistically significant ($p<0.05$) p-values bolded

Metal	Study area	Annual change (%)	95% CI	Log-lin. r^2 p	Non-param. tau p
THg	All areas	-1.30	-2.49 – 0.09	**p=0.035**	
	Northern Quark	-1.79	-3.90 – 0.37	p=0.102	p=0.13
	Lake Vanajanselkä	-0.83	-2.77 – 1.14	p=0.395	p=0.301
	Pristine SW Lake Area	-0.77	-2.87 – 1.38	p=0.468	p=0.403
MeHg	All areas	-1.93	-3.64 – -0.19	**p=0.030**	
	Northern Quark	-3.08	-6.14 – 0.09	p=0.056	p=0.086
	Lake Vanajanselkä	-1.22	-3.22 – 0.82	p=0.229	p=0.256
Se	All areas	-0.12	-0.66 – 0.42	p=0.661	
	Northern Quark	-0.43	-1.05 – 0.19	p=0.162	p=0.097
	Lake Vanajanselkä	-0.22	-0.69 – 0.26	p=0.357	p=0.577
Pb	All areas	-6.85	-8.18 – -5.50	**p<0.001**	
	Northern Quark	-7.49	-9.70 – -5.24	**p<0.001**	**p<0.001**
	Lake Vanajanselkä	-6.87	-8.53 – -5.18	**p<0.001**	**p<0.001**
Cr	All areas	-2.84	-4.02 – -1.64	**p<0.001**	
	Northern Quark	-3.35	-5.34 – -1.32	**p=0.002**	**p=0.006**
	Lake Vanajanselkä	-2.63	-4.21 – -1.04	**p=0.002**	**p=0.006**
Cu	All areas	-0.56	-1.46 – 0.35	p=0.224	
	Northern Quark	-1.30	-2.80 – 0.22	p=0.089	p=0.064
	Lake Vanajanselkä	-0.36	-1.54 – 0.84	p=0.543	p=0.345
Zn	All areas	-0.31	-1.06 – 0.46	p=0.426	
	Northern Quark	0.08	-0.84 – 1.01	p=0.861	p=0.628
	Lake Vanajanselkä	-0.26	-1.24 – 0.73	p=0.595	p=0.390

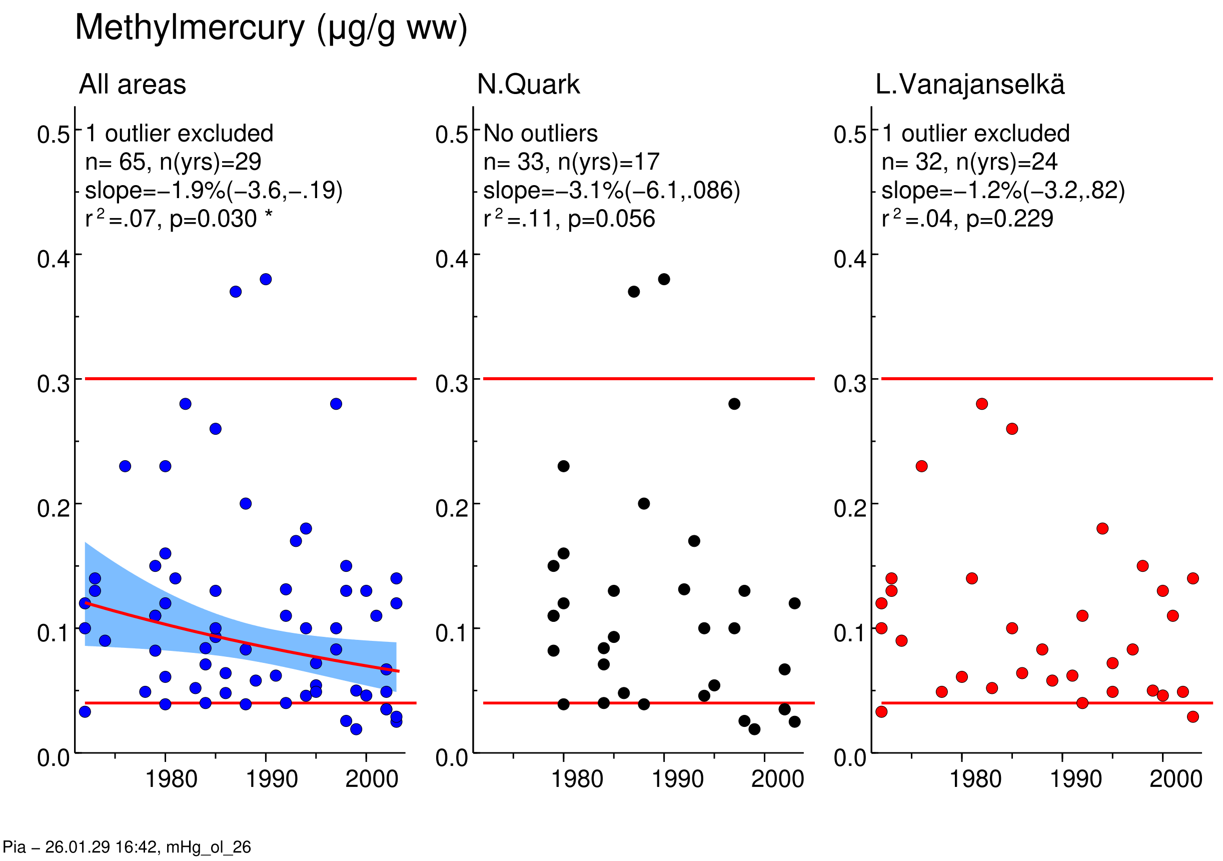

Online Resource Fig S1 Temporal trends of MeHg in osprey eggs in the whole dataset and in different study areas. Circles indicate individual data, and a red regression line is shown if $p<0.05$ (two-sided regression analysis). MeHg decreased significantly in all areas (annual decrease 1.93%). Shaded area is the 95% confidence band for the regression line and red horizontal lines indicate the toxicity reference values for low (0.04 µg/g ww) and moderate (0.3 µg/g ww) according to Ackerman et al., 2024

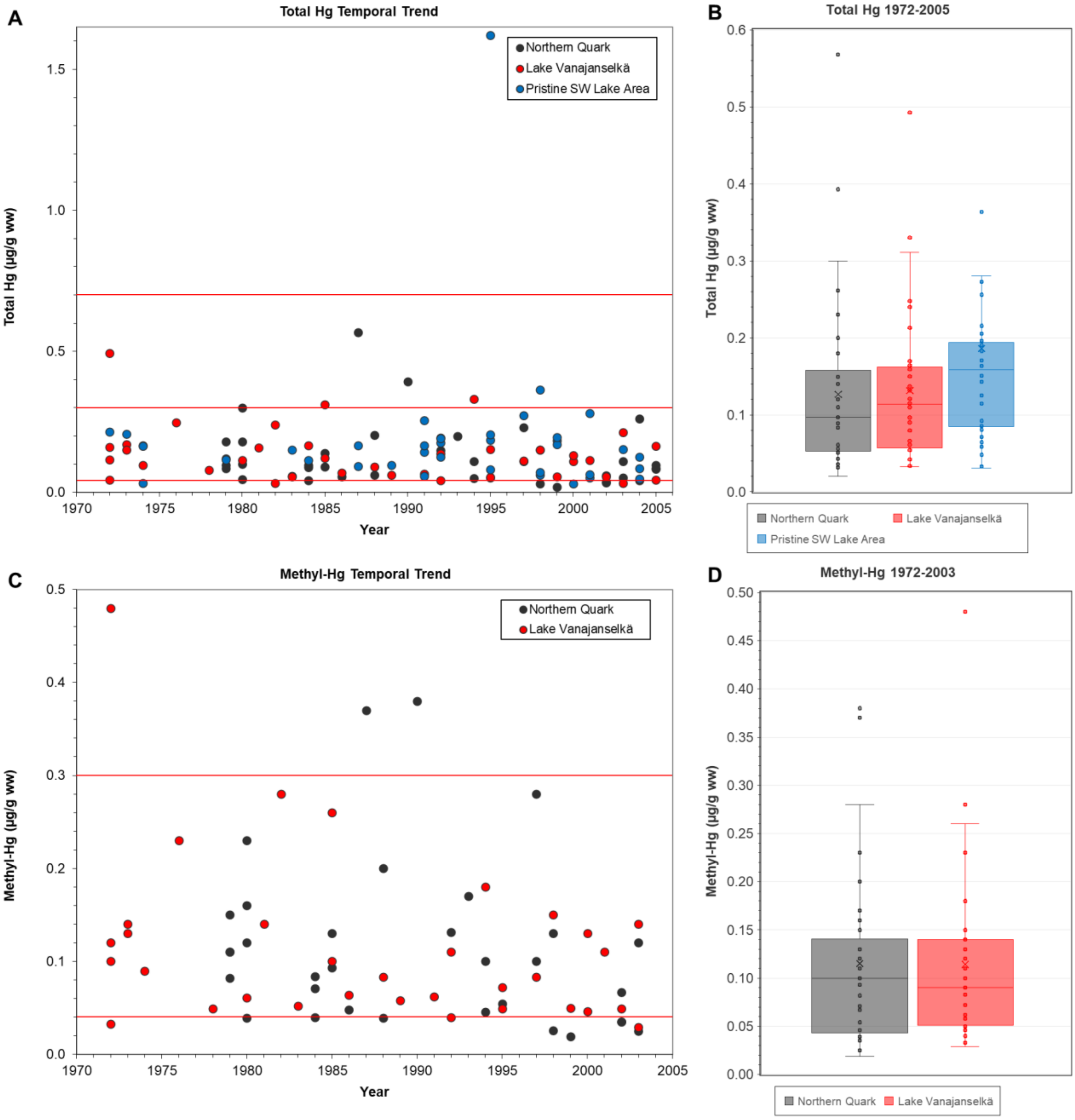

Online Resource Fig S2 Individual data and box plots on THg and MeHg of all samples. In left panel THg (A) in 1972-2005 and MeHg (C) in 1972-2003 temporal trends. The red horizontal lines indicate the toxicity reference values for low (0.04 µg/g ww), moderate (0.3 µg/g ww) and high injury (0.7 µg/g ww, A only) according to Ackerman et al., 2024. In right panel THg (B) and MeHg (D) means (x), medians (horizontal line), interquartile ranges, min and max datapoints excluding outliers (exceeding 1.5 times the interquartile range; bars) and individual values of all samples (1972-2017) in different study areas are shown

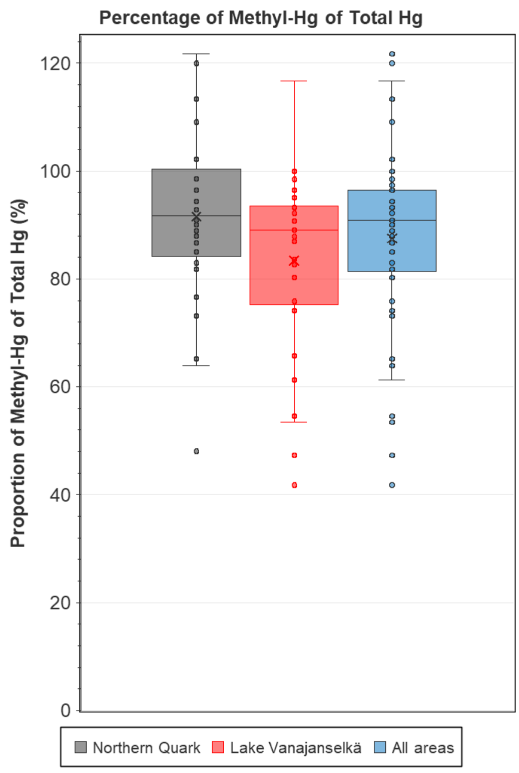

Online Resource Fig S3 Box plot on proportion of MeHg of THg in Northern Quark, Lake Vanajanselkä and all areas. Means (x), medians (horizontal line), interquartile ranges, min and max datapoints excluding outliers (exceeding 1.5 times the interquartile range; bars) and individual values of all samples (1972-2003) are shown

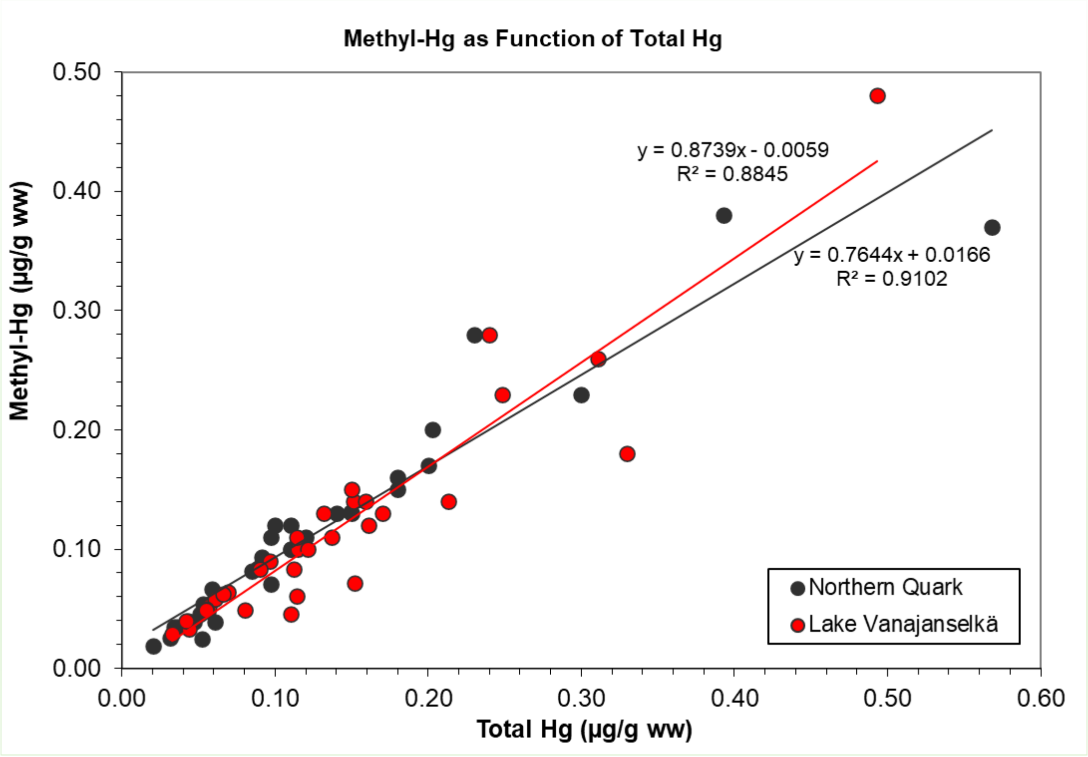

Online Resource Fig S4 Linear regression between MeHg and THg concentrations in Northern Quark and Lake Vanajanselkä

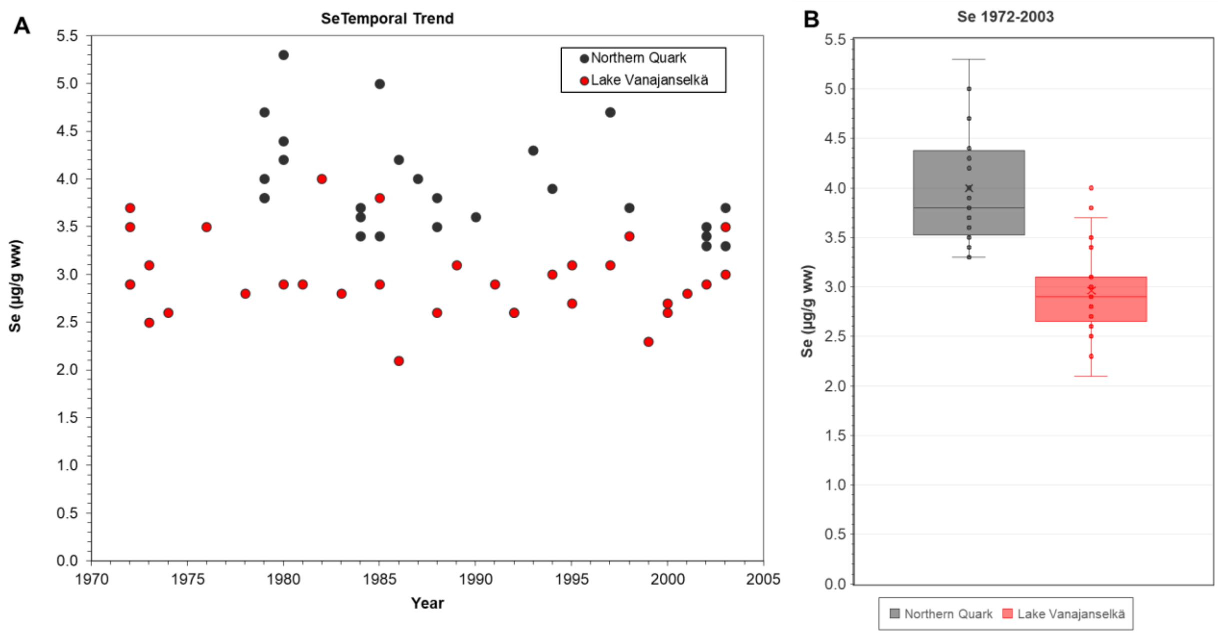

Online Resource Fig S5 Individual data and box plots on Se of all samples in Northern Quark and Lake Vanajanselkä. Individual data show no clear temporal trends (A). In panel B means (x), medians (horizontal line), interquartile ranges, min and max data points excluding outliers (exceeding 1.5 times the interquartile range; bars) and individual values of all samples (1972-2003) are shown. Se concentrations are higher in the Baltic Sea area of Northern Quark than in Lake Vanajanselkä. Se concentrations of the whole study period were significantly higher ($p<0.001$, t-test) in Northern Quark (mean 4.00, median 3.80 µg/g ww) than in Lake Vanajanselkä (mean 2.96, median 2.90 µg/g ww)

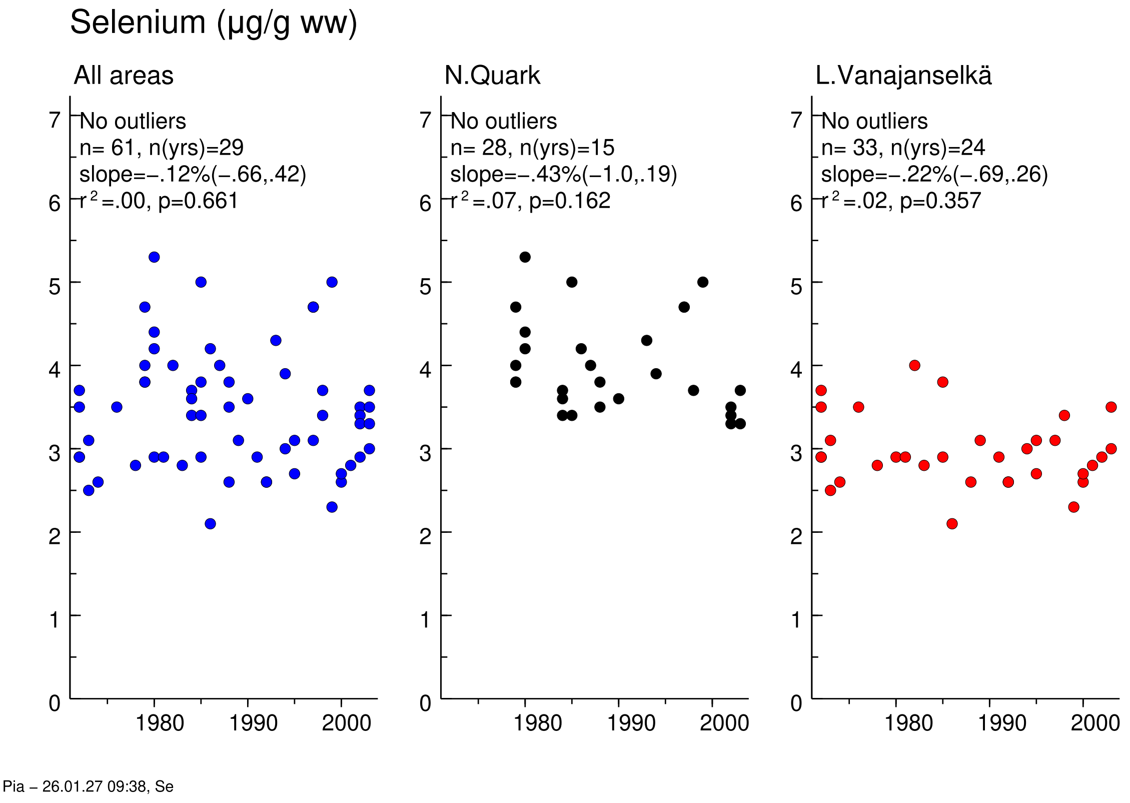

Online Resource Fig S6 Temporal trend of Se levels in osprey eggs in different study areas. Circles indicate individual data. There were no significant trends in the whole dataset or in the study areas

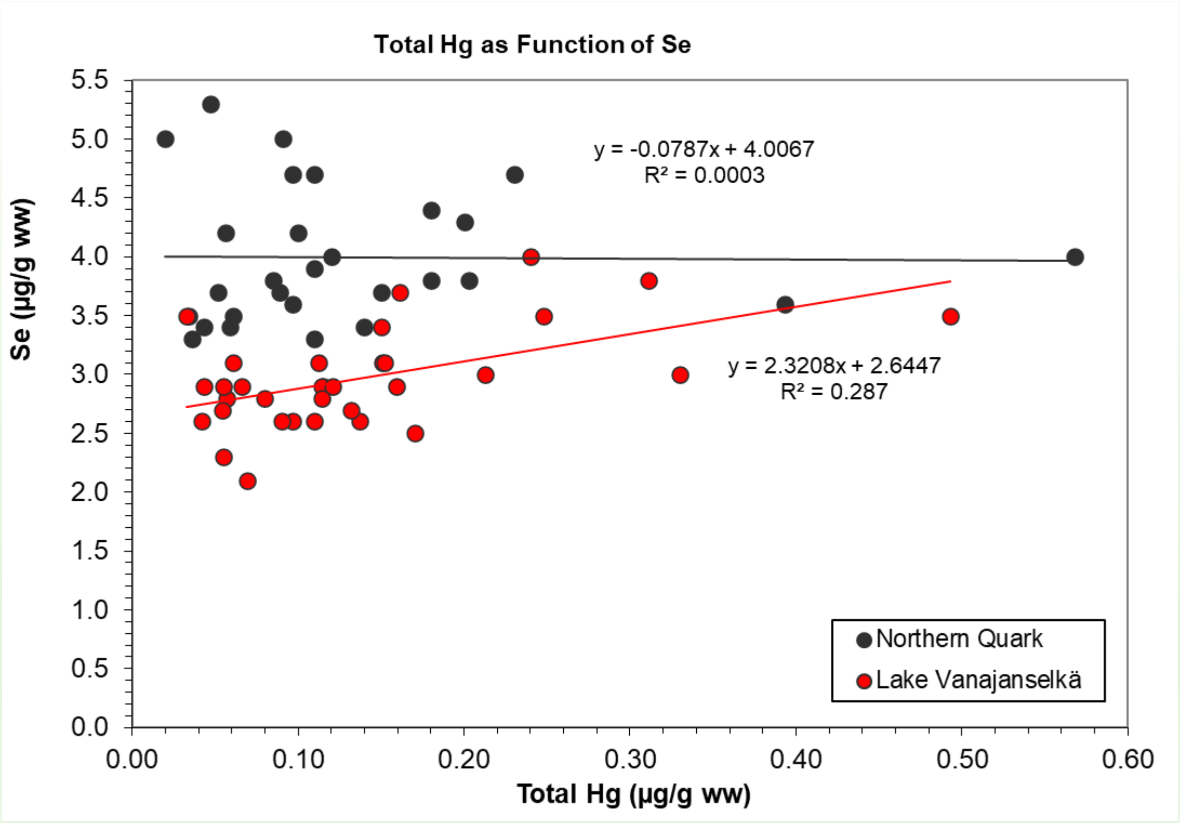

Online Resource Fig S7 Linear regression between Se and THg concentrations in Northern Quark and Lake Vanajanselkä

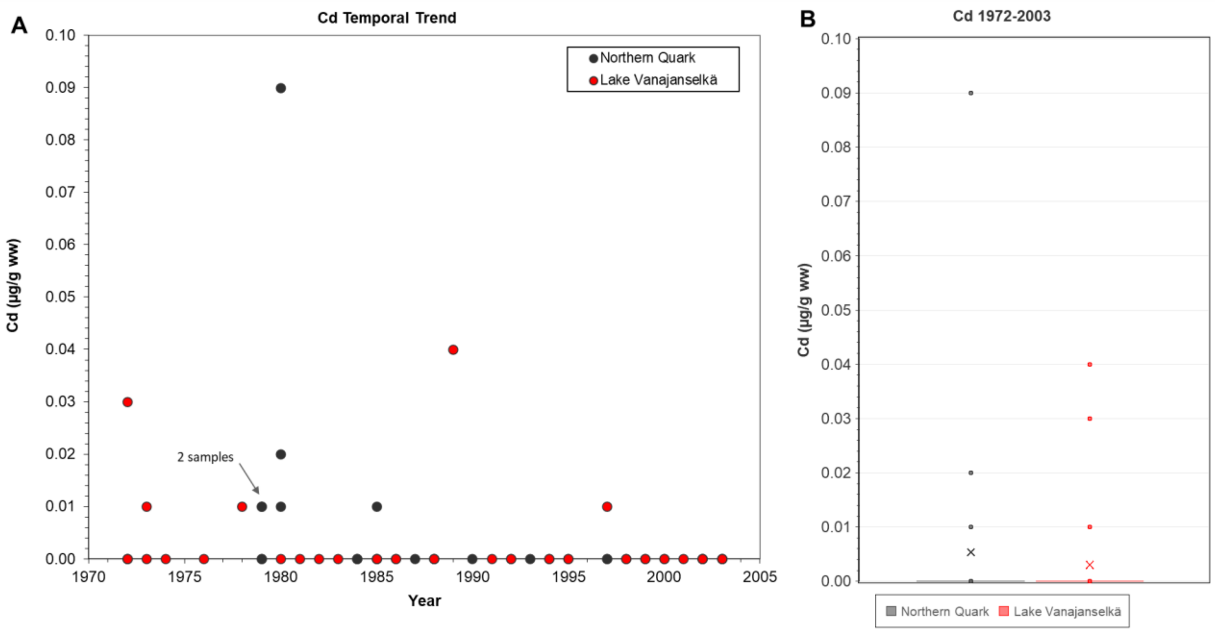

Online Resource Fig S8 Individual data and box plots on Cd of all samples in Northern Quark and Lake Vanajanselkä. Cd levels were quantified only in 5 samples out of 28 in Northern Quark and in 6 samples out of 33 in Lake Vanajanselkä (A). Cd was not detected after 1997. In panel B means (x), medians (horizontal line), interquartile ranges, min and max datapoints excluding outliers (exceeding 1.5 times the interquartile range; bars) and individual values of all samples (1972-2017) in different study areas are shown

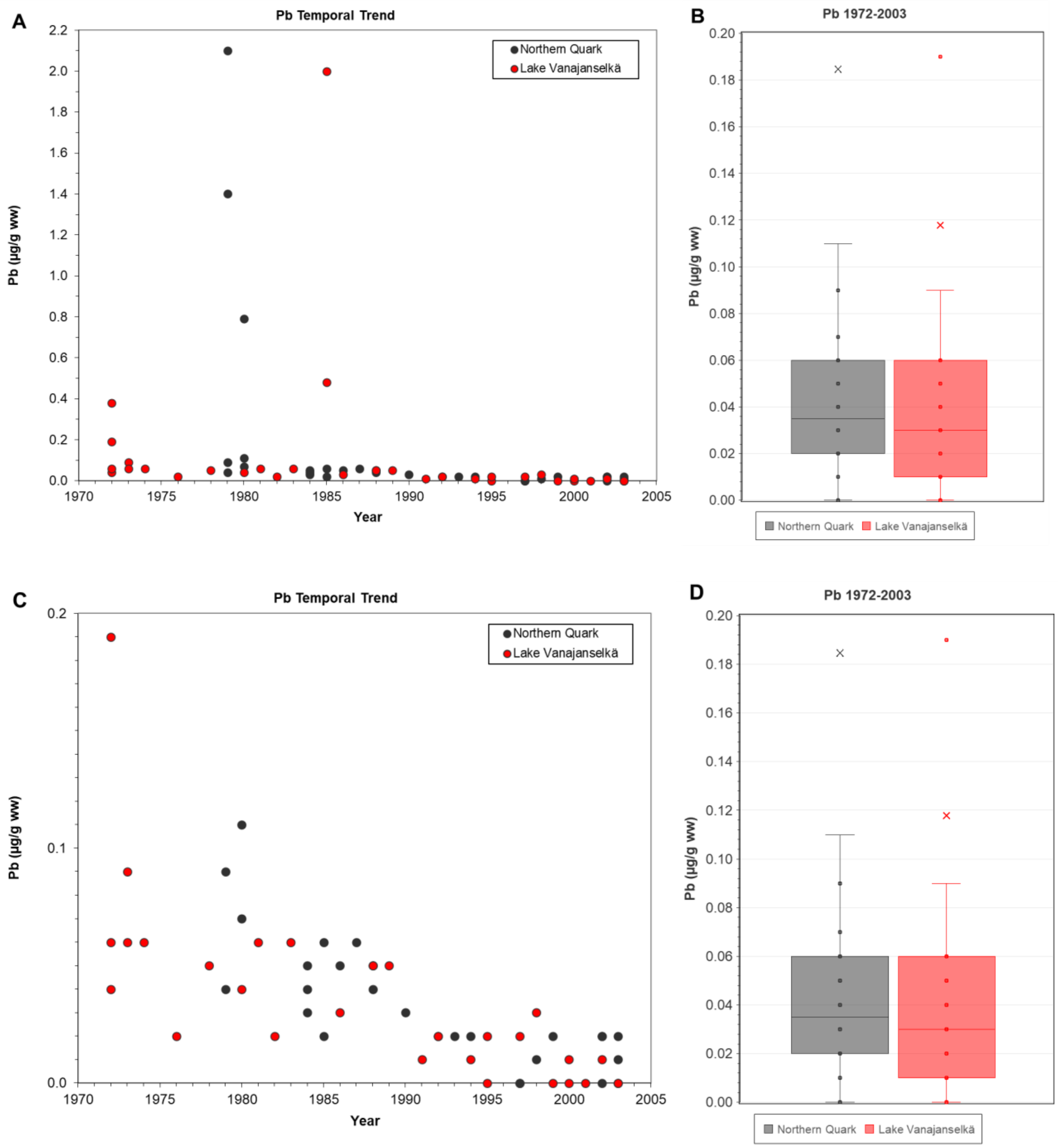

Online Resource Fig S9 Individual data and box plots on Pb of all samples in Northern Quark and Lake Vanajanselkä. Individual data show decreasing temporal trends (A). In panel B means (x), medians (horizontal line), interquartile ranges, min and max data points excluding outliers (exceeding 1.5 times the interquartile range; bars) and individual values of all samples (1972-2003) are shown. In panel C outliers shown in panel A are excluded

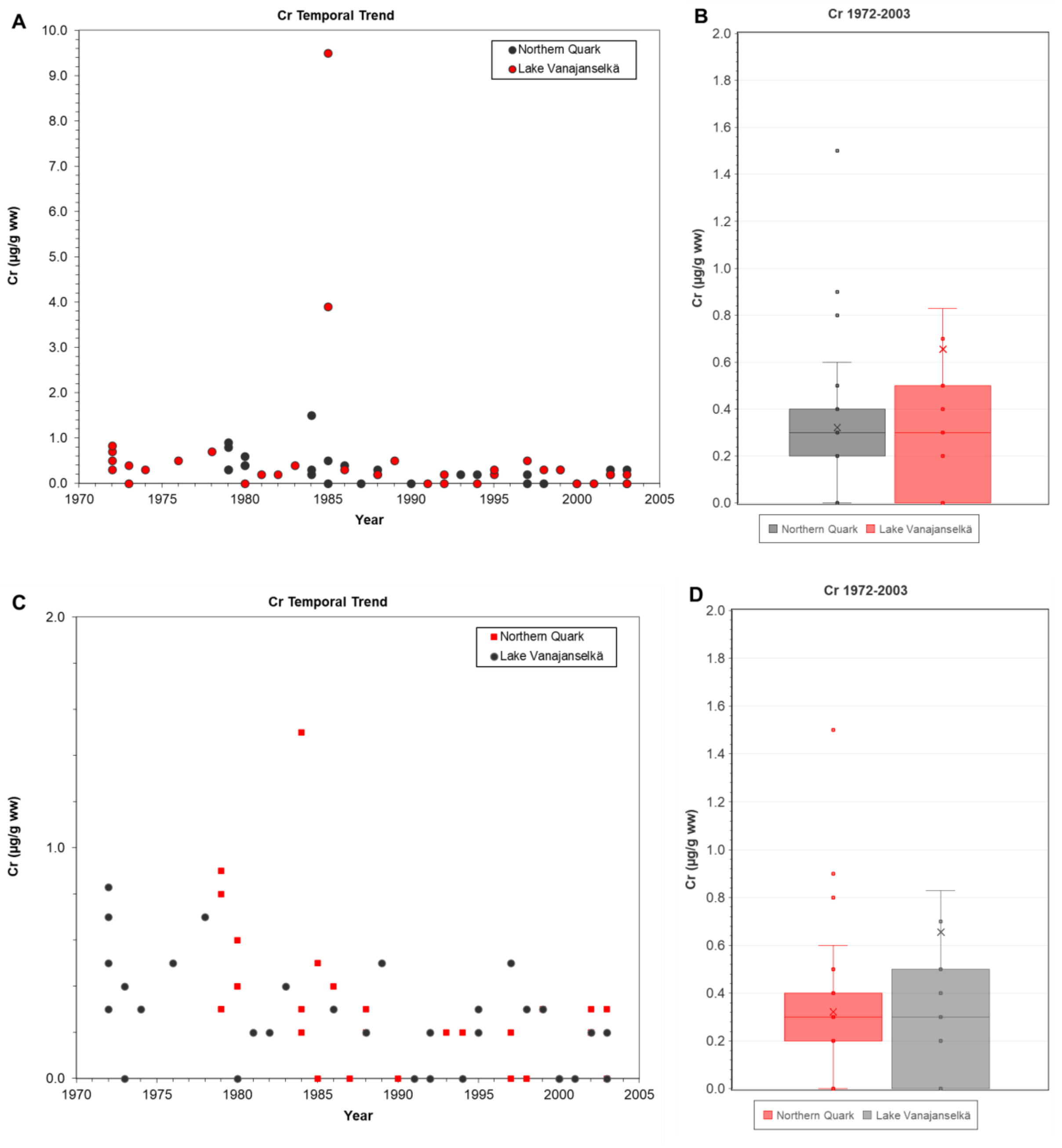

Online Resource Fig S10 Individual data and box plots on Cr of all samples in Northern Quark and Lake Vanajanselkä. Individual data show slightly decreasing temporal trends (A). In panel B means (x), medians (horizontal line), interquartile ranges, min and max data points excluding outliers (exceeding 1.5 times the interquartile range; bars) and individual values of all samples (1972-2003) are shown. In panel C outliers shown in panel A are excluded

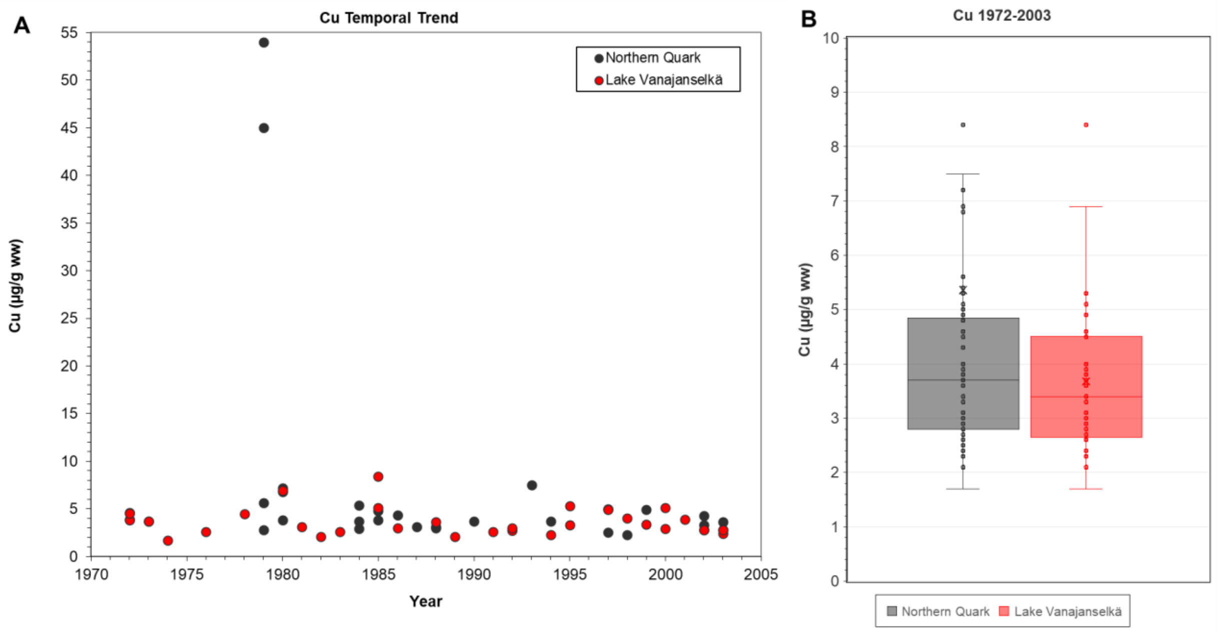

Online Resource Fig S11 Individual data and box plots on Cu of all samples in Northern Quark and Lake Vanajanselkä. Individual data show slightly decreasing temporal trends (A). In panel B means (x), medians (horizontal line), interquartile ranges, min and max data points excluding outliers (exceeding 1.5 times the interquartile range; bars) and individual values of all samples (1972-2003) are shown

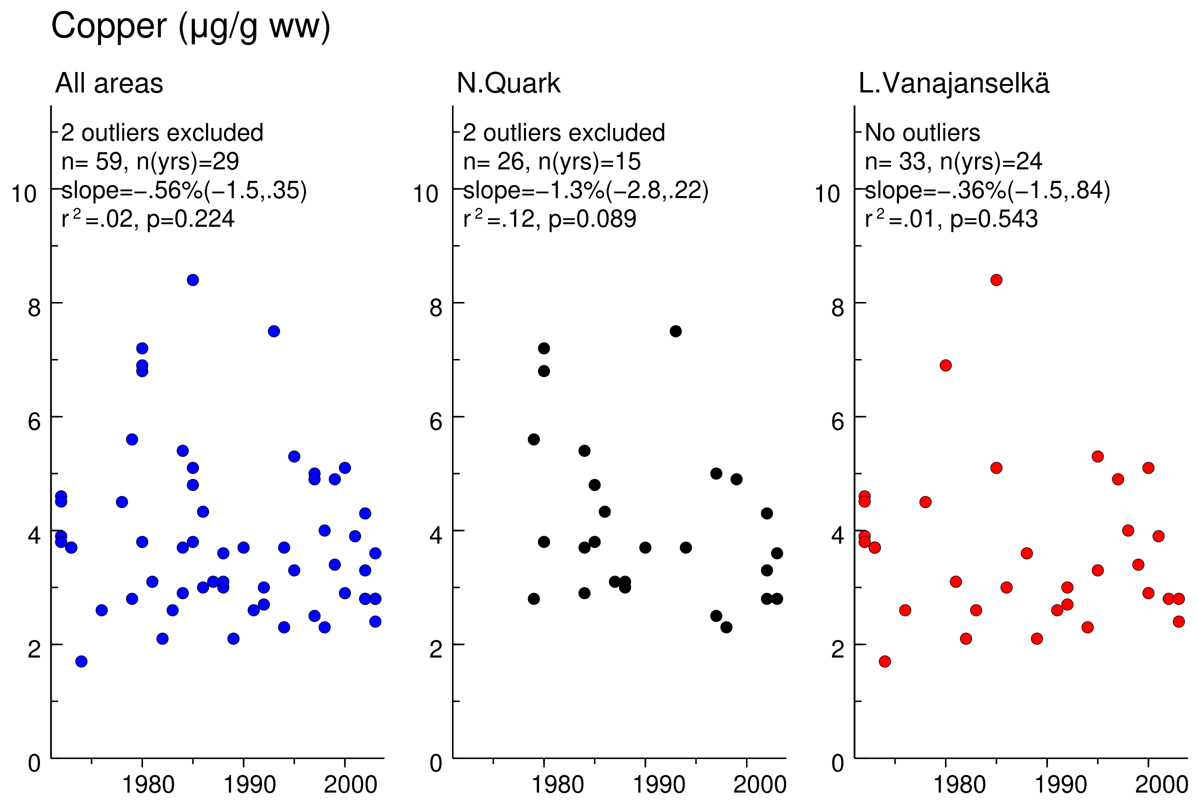

Pia – 26.01.27 09:38, Cu

Online Resource Fig S12 Temporal trend of Cu levels in osprey eggs in the whole dataset and in both study areas. Circles indicate individual data. The trends were nonsignificant

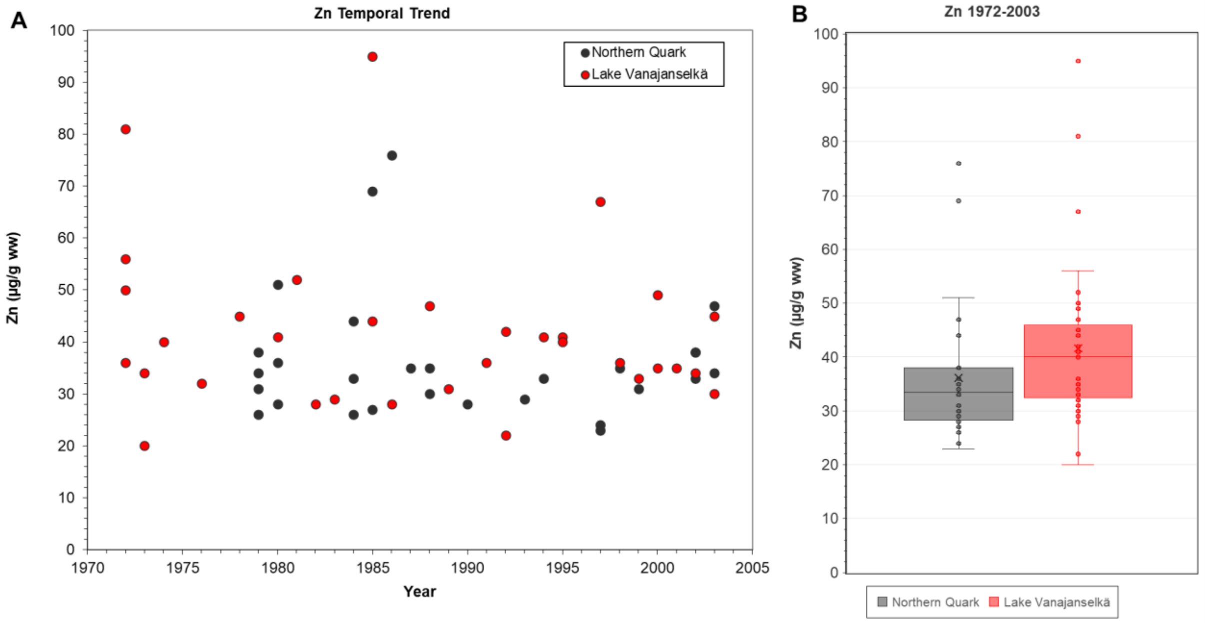

Online Resource Fig S13 Individual data and box plots on Zn of all samples in Northern Quark and Lake Vanajanselkä. Individual data show no temporal trends (A). In panel B means (x), medians (horizontal line), interquartile ranges, min and max data points excluding outliers (exceeding 1.5 times the interquartile range; bars) and individual values of all samples (1972-2003) are shown. Zn concentrations are higher in Lake Vanajanselkä than in Northern Quark

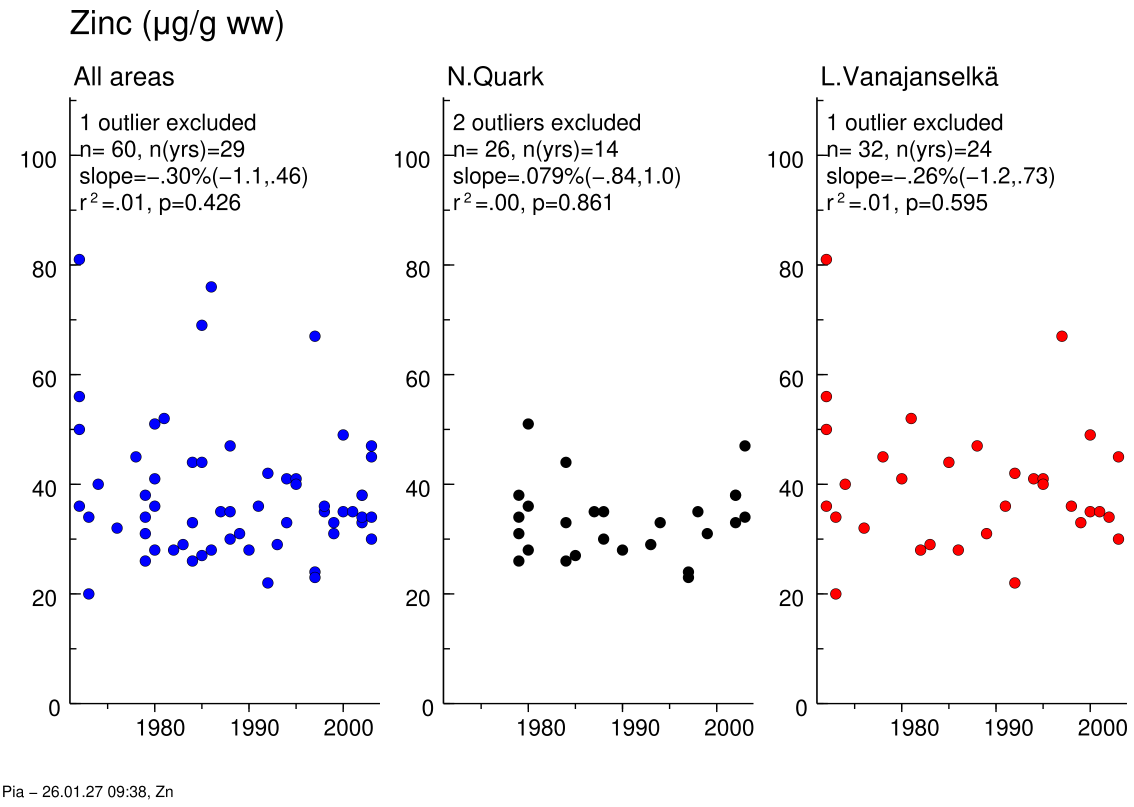

Online Resource Fig S14 Temporal trend of Zn levels in osprey eggs in different study areas. Blue circles indicate individual data. The trends were nonsignificant in the whole dataset or in the study areas

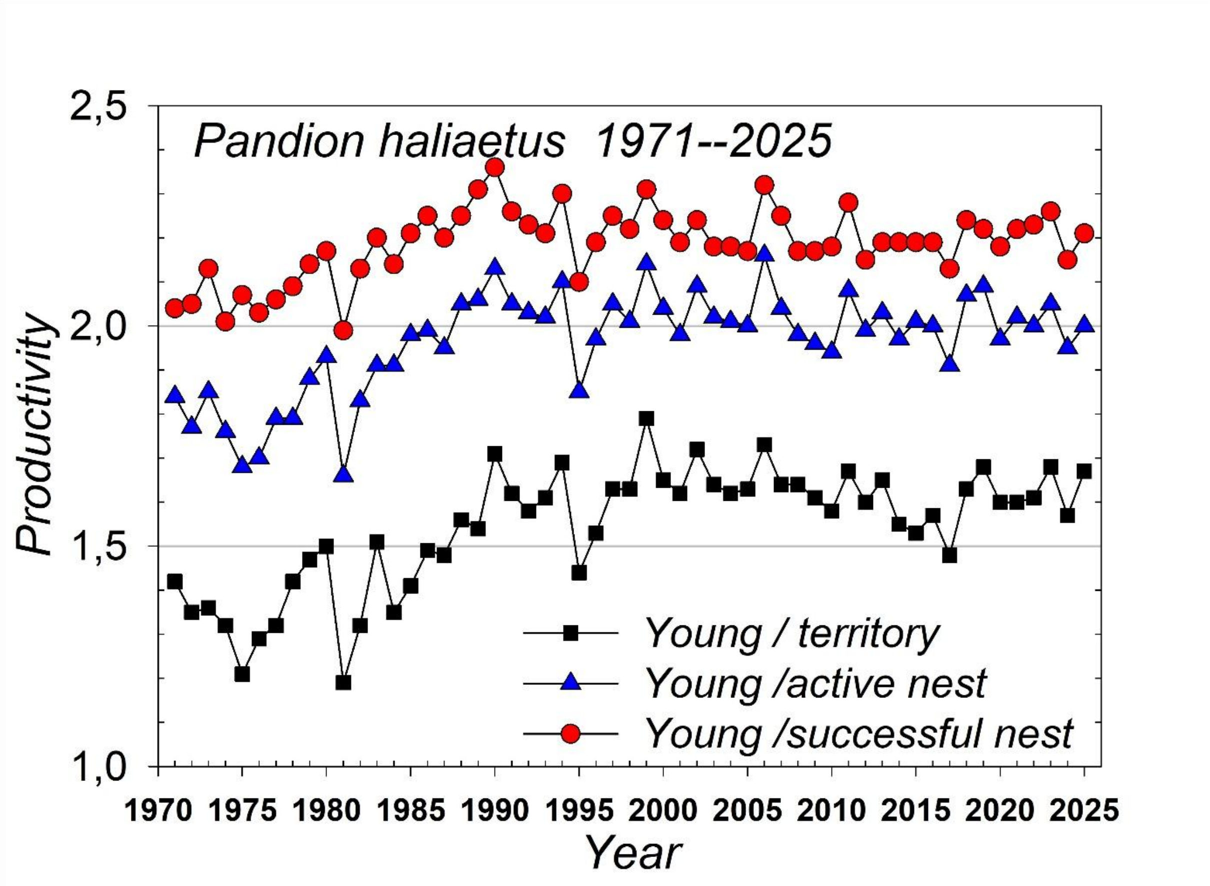

Online Resource Fig S15 Average productivity of the Finnish ospreys in 1971–2025. Black square = chicks / occupied territory, blue triangle = chicks / active nest, red dot = chicks / successful nest. Data from the database of the Finnish Museum of Natural History (Saurola, 2025)
